# Alteration of *S*‐adenosylhomocysteine levels affects lignin biosynthesis in switchgrass

**DOI:** 10.1111/pbi.12935

**Published:** 2018-06-06

**Authors:** Zetao Bai, Tianxiong Qi, Yuchen Liu, Zhenying Wu, Lichao Ma, Wenwen Liu, Yingping Cao, Yan Bao, Chunxiang Fu

**Affiliations:** ^1^ Shandong Provincial Key Laboratory of Energy Genetics Qingdao Institute of Bioenergy and Bioprocess Technology Chinese Academy of Sciences Qingdao Shandong China; ^2^ Key Laboratory of Biofuels Qingdao Institute of Bioenergy and Bioprocess Technology Chinese Academy of Sciences Qingdao Shandong China; ^3^ Qingdao Engineering Research Center of Biomass Resources and Environment Qingdao Institute of Bioenergy and Bioprocess Technology Chinese Academy of Sciences Qingdao Shandong China

**Keywords:** cystathionine γ‐synthase, lignin biosynthesis, methionine metabolism, *Panicum virgatum* L. (switchgrass), *S*‐adenosylhomocysteine, *S*‐adenosylhomocysteine hydrolase, *S*‐adenosylmethionine

## Abstract

Methionine (Met) synthesized from aspartate is a fundamental amino acid needed to produce *S*‐adenosylmethionine (SAM) that is an important cofactor for the methylation of monolignols. As a competitive inhibitor of SAM‐dependent methylation, the effect of *S*‐adenosylhomocysteine (SAH) on lignin biosynthesis, however, is still largely unknown in plants. Expression levels of *Cystathionine* γ*‐synthase* (*PvCGS*) and *S‐adenosylhomocysteine hydrolase 1* (*PvSAHH1*) were down‐regulated by RNAi technology, respectively, in switchgrass, a dual‐purpose forage and biofuel crop. The transgenic switchgrass lines were subjected to studying the impact of SAH on lignin biosynthesis. Our results showed that down‐regulation of *PvCGS* in switchgrass altered the accumulation of aspartate‐derived and aromatic amino acids, reduced the content of SAH, enhanced lignin biosynthesis and stunted plant growth. In contrast, down‐regulation of *PvSAHH1* raised SAH levels in switchgrass, impaired the biosynthesis of both guaiacyl and syringyl lignins and therefore significantly increased saccharification efficiency of cell walls. This work indicates that SAH plays a crucial role in monolignol methylation in switchgrass. Genetic regulation of either *PvCGS* or *PvSAHH1* expression in switchgrass can change intracellular SAH contents and SAM to SAH ratios and therefore affect lignin biosynthesis. Thus, our study suggests that genes involved in Met metabolism are of interest as new valuable targets for cell wall bioengineering in future.

## Introduction

Plant cell walls store about 30%–40% of the annually fixed terrestrial organic carbon in the biosphere and are a rich source of fermentable sugars and biopolymers (Zhang and Liu, 2015). Lignin is a major component of vascular plant cell walls (Boerjan *et al*., 2003). As a complex phenolic polymer, lignin consists mainly of hydroxyphenyl (H), guaiacyl (G) and syringyl (S) units that are formed through an oxidative polymerization of *p*‐coumaryl, coniferyl and sinapyl alcohols, respectively (Boerjan *et al*., 2003). Lignin linked to carbohydrate components provides rigidity, strength and hydrophobicity to cell walls (Boerjan *et al*., 2003). Thus, the major biological function of lignin is mechanical support, water conductivity and protection against plant pathogens. Lignin, however, has a remarkable impact on efficient pulping, forage digestion and biofuel production of lignocellulosic biomass (Ragauskas *et al*., 2006). Previous studies have suggested that the saccharification efficiency of cell walls is negatively correlated with lignin content (Carroll and Somerville, 2009; Chen and Dixon, 2007; Pilate *et al*., 2002). Thus, genetic manipulation of lignin is of great interest in the pulp and paper industry, agriculture–livestock industry and environmental protection (Bedon and Legay, 2011).

During recent decades, the structural genes and their transcriptional regulators in the lignin biosynthetic pathway have been genetically modified in many dicot and monocot plant species. The number of target genes, however, is still not sufficient to meet the needs of lignin engineering. Recently, two studies demonstrate that methylenetetrahydrofolate reductase (MTHFR) and folylpolyglutamate synthase (FPGS) can significantly affect lignin biosynthesis, which may shed light on a new regulatory mechanism of lignin biosynthesis (Li *et al*., 2015; Tang *et al*., 2014). MTHFR and FPGS are involved in plant one‐carbon (C1) metabolism that is essential in all organisms for the biosynthesis of methionine (Met), *S*‐adenosylmethionine (SAM), *S*‐adenosylhomocysteine (SAH), homocysteine (Hcy) and other methylated compounds (Hanson and Roje, 2001). Generally, C1 metabolism includes tetrahydrofolate (THF) and methionine (Met) cycles. The former is responsible for the transfer of C1 units, while the latter is for the synthesis and recycling of SAM, the activated form of Met. In the lignin biosynthetic pathway, caffeoyl CoA *O*‐methyltransferase (CCoAOMT) and caffeic acid *O*‐methyltransferase (COMT) are required to methylate 3‐ and 5‐hydroxyl groups of the aromatic ring, respectively, using SAM as a cofactor and ultimately lead to the biosynthesis of G and S lignins (Boerjan *et al*., 2003; Roje, 2006). Moreover, many C1 metabolism genes exhibit high expression levels in well‐lignified tissues in plants, implying a potential coordination between C1 metabolism and lignin biosynthesis (Scully *et al*., 2018; Srivastava *et al*., 2015; Villalobos *et al*., 2012).

**Figure 1:**
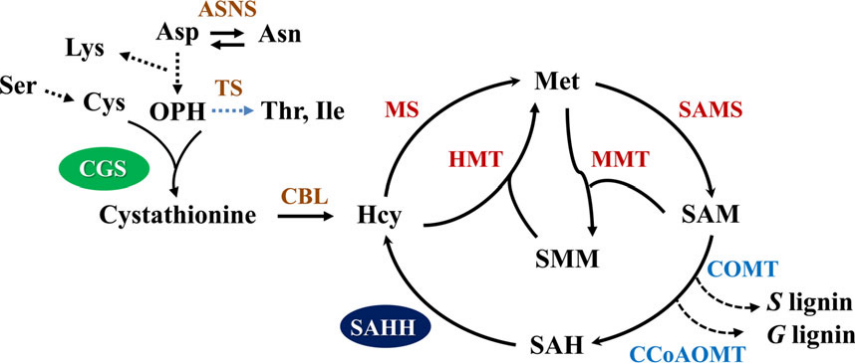
Biosynthesis of methionine in higher plants. The key enzymes involved in methionine metabolism and monolignol methylation and the Asp‐derived amino acids potentially affected by CGS disruption are indicated. One metabolic step is represented by full arrows, while several metabolic steps are represented by dashed arrows. Asp, Aspartate; Asn, asparagine; Lys, lysine; Ser, serine; Cys, cysteine; OPH,* O*‐phosphohomoserine; Thr, threonine; Ile, isoleucine; Hcy, homocysteine; Met, methionine; SMM,* S*‐methylmethionine; SAM,* S*‐adenosylmethionine; SAH,* S*‐adenosylhomocysteine; G, guaiacyl; S, syringyl; ASNS, asparagine synthetase; CGS, cystathionine γ‐synthase; TS, threonine synthase; CBL, cystathionine β‐lyase; MS, methionine synthase; SAMS,* S*‐adenosylmethionine synthase; HMT, homocysteine *S*‐methyltransferase; MMT, methionine *S*‐methyltransferase; SAHH,* S*‐adenosylhomocysteine hydrolase; COMT, caffeic acid *O*‐methyltransferase; CCoAOMT, caffeoyl CoA *O*‐methyltransferase.

Cystathionine γ*‐*synthase (CGS) is the first committed enzyme for the biosynthesis of Met that can be metabolized to SAM (Figure 1). This enzyme combines *O*‐phosphohomoserine (OPH) derived from aspartate (Asp) with the thiol group of cysteine (Cys) to form cystathionine (Galili *et al*., 2016). Threonine synthase (TS), however, competes with CGS for OPH that is metabolized towards threonine (Thr) and isoleucine (Ile) (Amir *et al*., 2002). Therefore, the biosynthesis of Met is controlled by both CGS and TS. Previous studies have indicated that the overexpression of *CGS* dramatically elevates the concentrations of Met and *S*‐methylmethionine (SMM) in *Arabidopsis* without impacting plant growth and development (Gakière *et al*., 2002; Kim *et al*., 2002). By contrast, down‐regulation of *AtCGS* in *Arabidopsis* affects Met accumulation slightly in spite of a substantial increase in OPH and a severe growth retardation (Gakière *et al*., 2000; Kim and Leustek, 2000). Most strikingly, either up‐regulating or down‐regulating *CGS* in the seeds of *Arabidopsis* can significantly increase the concentrations of Met and result in almost similar effects on the biosynthesis of other amino acids including Thr, Ile and phenylalanine (Phe) (Cohen *et al*., 2014, 2017a; Kim *et al*., 2002). In addition, the overexpression of Met‐insensitive form of *AtCGS* increases Met levels dramatically in other dicot species including tobacco, alfalfa, soybean and azuki bean (Avraham *et al*., 2005; Hacham *et al*., 2008; Hanafy *et al*., 2013). However, the overexpression of *StCGS* has no effect on the biosynthesis of Met in potato (Kreft *et al*., 2003). Furthermore, down‐regulation of *CGS* in potato caused a significant reduction in free Met levels, but no visible phenotypic changes (Kreft *et al*., 2003). Thus, the effect of *CGS* alteration on plant growth and development, Met and other related amino acid metabolism still remains largely unknown.

The precursors of G and S lignins are the abundant methylated products derived from phenylpropanoid and SAM biosynthetic pathways. Previous research has shown that a single‐point mutation in the *S‐adenosylmethionine synthase 3* (*SAMS3*) can significantly impair both SAM and lignin accumulation in *Arabidopsis* (Shen *et al*., 2002). Moreover, disruption of THF cycle genes such as *MTHFR* and *FPGS* also affect lignin biosynthesis substantially (Li *et al*., 2015; Srivastava *et al*., 2015; Tang *et al*., 2014). These studies suggest that C1 metabolism may control lignin biosynthesis through regulating the metabolism of SAM, the methyl group donor involved in the methylation of monolignols. In addition, as the demethylation product of SAM, SAH competes with SAM binding, which inhibits the enzymatic activities of methyltransferases (Keating *et al*., 1991; Nguyen *et al*., 2001). An *S*‐adenosylhomocysteine hydrolase (SAHH) is responsible for SAH hydrolysis that is crucial to maintain SAH levels in plants (Hanson and Roje, 2001). Disruption of *SAHH1* in *Arabidopsis* impacts plant growth and development obviously (Rocha *et al*., 2005). However, the role of SAHH in lignin biosynthesis has yet to be investigated.

Switchgrass (*Panicum virgatum* L.), a dual‐purpose forage and biofuel crop, is a perennial C4 tall grass native to North America (McLaughlin and Kszos, 2005). In this research, we identified the *CGS* gene from switchgrass and studied the effect of *PvCGS* on the Met cycle, Asp family amino acids and lignin biosynthesis. Our results showed that severe down‐regulation of *PvCGS* in switchgrass resulted in growth stunting. Moreover, down‐regulation of *PvCGS* increased lignin biosynthesis through reducing SAH contents and inducing phenylalanine and tyrosine accumulation in switchgrass. Furthermore, elevating SAH levels by down‐regulation of *PvSAHH1* in switchgrass enhanced its inhibition on monolignol methylation and therefore impaired the biosynthesis of both G and S lignins. Lignin alteration in SAHH‐RNAi transgenic switchgrass plants improved saccharification efficiency of cell walls without biomass penalty, thus providing a potential for improving biofuel production and forage digestibility in future.

## Results

### Expression pattern of *PvCGS* positively correlated with those of *PvSAMS* and *PvSAHH1* in the process of internode lignification

**Figure 2:**
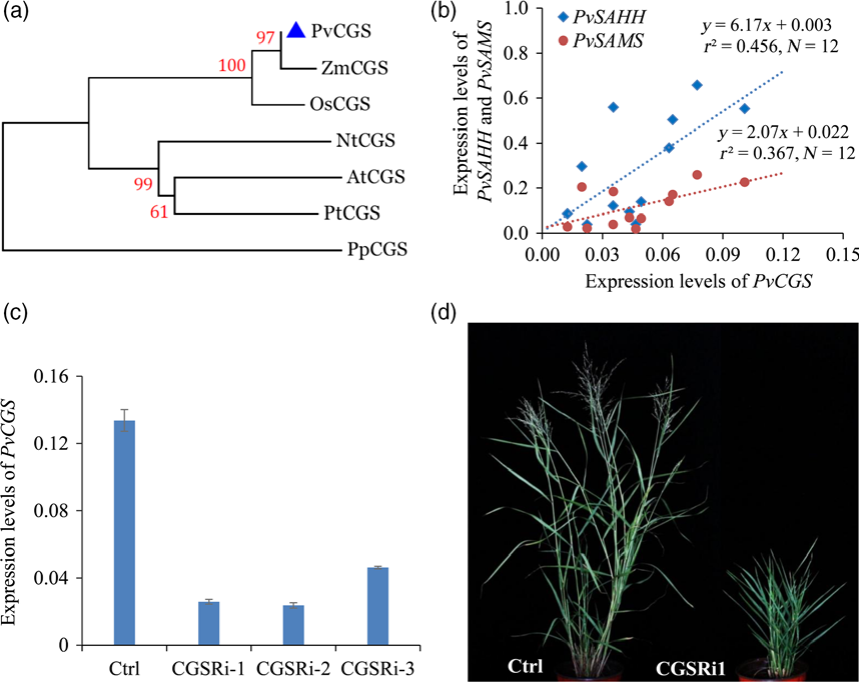
Characterization of CGS‐RNAi transgenic switchgrass plants. (a) Phylogenetic tree of the CGSs in dicot and monocot plant species. A maximum likelihood tree was constructed in PhyML version 3.0 on the basis of multiple alignments of the deduce protein sequences from *Panicum virgatum* (PvCGS, Pavir. 9NG556700), *Zea mays* (ZmCGS, GRMZM2G113873), *Oryza sativa* (OsCGS, LOC_Os03 g25940), *Arabidopsis thaliana* (AtCGS, AT3G01120), *Nicotiana tobacum* (NtCGS, mRNA_86886_cds), *Populus trichocarpa* (PtCGS, POPTR_0017s12240) and *Physcomitrella patens* (PpCGS, Pp1s49_246V6). Bootstrap values (>50%) based on 1000 replications are indicated at nodes. The above sequence data are retrieved from Phytozome and Sol Genomics Network. (b) Correlationships between expression levels of *PvCGS* and *PvSAMS*/*PvSAHH*. The expression levels of *PvCGS*,* PvSAHH* and *PvSAMS* were measured by quantitative real‐time PCR. Switchgrass *UBQ* was used as the reference for normalization. The correlations between the expression levels of *PvCGS* and *PvSAHH/PvSAMS* were statistically significant (*P *<* *0.05). Internodes, leaf sheaths and leaf blades of internode 2 (I2), internode 3 (I3) and internode 4 (I4) at E4 stage and the I2 internodes at E2, E3 and E4 stages were collected from wild‐type switchgrass plants. (c) Quantitative real‐time PCR analysis of *PvCGS* transcript abundance in the CGS‐RNAi transgenic switchgrass lines. Switchgrass *UBQ* was used as the reference for normalization. Stems at the E4 stage were collected. Value are mean ± SE (*n* = 3). (d) Morphological characterization of transgenic switchgrass plants with down‐regulation of *CGS*. The control plants (Ctrl) for CGS‐RNAi transgenic lines were generated with pANIC8B empty vector.

To study the function of *CGS* in switchgrass, we first identified *PvCGS* sequences from *Panicum virgatum* v4.1 genome database (Phytozome). The switchgrass genome assembly contains a pair of *PvCGS* genes that exist on chromosome 9 and share over 99% sequence identities to each other. The orthologs of *PvCGS* identified from two monocots (*Zea mays* and *Oryza sativa*), three dicots (*Arabidopsis thaliana*,* Nicotiana tabacum* and *Populus trchocarpa*) and one moss (*Physcomitrella patens*) were employed for the phylogenetic relationship analysis. The phylogenetic tree shows that PvCGS is clustered together in a group with those from monocot species (Figure 2a). Moreover, alignment of the CGS amino acid sequences reveals high similarity between PvCGS and AtCGS (Figure S1).

The downstream enzymes of CGS in Met metabolism, SAMS and SAHH, are responsible for the biosynthesis of SAM and SAH, which are involved in the methylation of lignin monomers (Figure 1). To study the potential effects of these C1 metabolism genes on lignin biosynthesis, the expression levels of *PvCGS*,* PvSAMS* and *PvSAHH1* in the process of internode lignification were detected by qRT–PCR. The tillers at three elongation stages (E2, E3 and E4) and one reproductive stage (R1) are associated with a significantly progressive lignification of cell walls. Therefore, we collected the second internode (I2) from the corresponding tillers at the above stages. The successive internodes (I2‐4) and their corresponding leaf sheaths and leaf blades were dissected from the tillers harvested at the E4 stage. Our results revealed that the expression levels of *PvCGS* positively correlated with those of *PvSAMS* (*r* ^2* *^= 0.367, *P *<* *0.05) and *PvSAHH1* (*r* ^2^ = 0.456, *P *<* *0.05) (Figure 2b, Table S1). Therefore, the full‐length cDNA sequence of *PvCGS* (Pavir.9NG556700) was isolated from switchgrass for investigating its function in Met metabolism and lignin biosynthesis.

### Down‐regulation of *PvCGS* affected switchgrass growth and development

To examine the function of CGS in switchgrass, we produced CGS‐RNAi transgenic switchgrass plants using a single genotypic embryogenic callus line. The control plants were produced with pANIC8B empty vector that was employed as the backbone for constructing CGS‐RNAi vector. Three independent positive transgenic switchgrass lines in which the transcript abundance of *PvCGS* was dramatically down‐regulated were selected for further studies (Figure 2c). Morphological characterization of the transgenic lines showed that substantial down‐regulation of *PvCGS* in switchgrass resulted in severe growth stunting and distinct delay in flowering (Figure 2d). Furthermore, we studied the detailed phenotype changes in CGS‐RNAi transgenic switchgrass lines including plant height, internode length, internode diameter, leaf sheath length, leaf blade length and width, and flowering time (Table S2). The plant height of transgenic plants was approximately 62% shorter than that of control plants. The number of internodes was not altered in the transgenic plants although approximately 57% reduction in the internode length was observed. Moreover, no flowering was observed in the transgenic lines in our glasshouse conditions with 16‐h light (Figure 2d, Table S2).

### Down‐regulation of *PvCGS* affected Met cycling and amino acid accumulation

**Figure 3:**
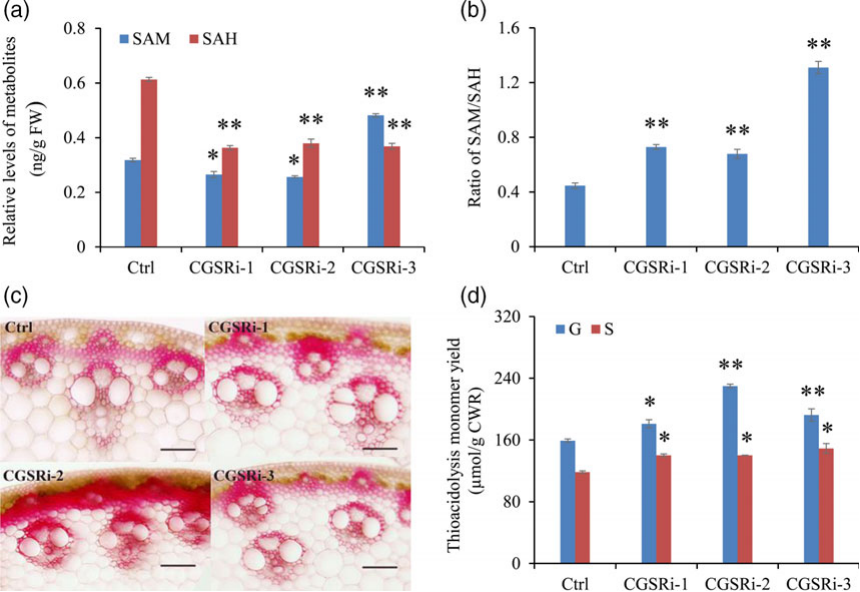
The effects of *CGS* down‐regulation on C1 metabolites and lignin biosynthesis in switchgrass. (a) The contents of SAM and SAH in control and transgenic switchgrass plants. (b) The ratios of SAM/SAH in control and transgenic switchgrass plants. The control plants (Ctrl) for CGS‐RNAi transgenic lines were generated with pANIC8B empty vector. (c) Cross sections of the I2 internodes from transgenic switchgrass plants. The control plants were generated with pANIC8B empty vector. Stem at E4 stage was collected, and the different internodes were separated. Cross sections from three independent I2 internodes were used for histochemical assay. Two technical replicates were conducted. Bars = 0.1 mm. (d) G and S lignin monomer yield of the CGS‐RNAi transgenic lines. The control plants (Ctrl) were generated with pANIC8B empty vector. Stems at E4 stage were collected. FW, fresh weight; CWR, cell wall residue. Value are mean ± SE (*n* = 3). One or two asterisks indicate significance corresponding to *P *<* *0.05 or 0.01 (one‐way ANOVA Dunnett's test).

To explore the effect of *PvCGS* down‐regulation on Met metabolism, we measured the contents of intermediates in the Met cycle including SAM, SAH, Hcy and Met. As SAM, SAH and Hcy are fairly labile in tissue extracts, we determined their relative levels by ELISA method. Our results showed that the contents of SAH were reduced by 38.1%–40.8% in all selected transgenic lines compared with control plants. In contrast, large variations were detected in the levels of SAM and Hcy in the transgenic lines (Figure 3a, Figure S2). Finally, the dramatic reduction in SAH levels raised the ratios of SAM/SAH in all transgenic lines (Figure 3b). Furthermore, we studied the effect of *PvCGS* down‐regulation on the accumulation of free Met and other amino acids, especially the Asp‐derived ones. We randomly selected line CGSRi‐1, combined with line CGSRi‐3, for further amino acid profiling analysis. Our results showed that the contents of 19 of 26 amino acids detected in switchgrass stems were significantly altered in CGS‐RNAi transgenic switchgrass lines (Table 1). Surprisingly, Met levels were little changed in the transgenic lines, although the levels of other Asp‐derived amino acids were substantially increased, including Asp, Thr, Ile, asparagine (Asn) and lysine (Lys) (Table 1). Moreover, the transgenic lines accumulated 2.9‐ and 2.5‐fold more phenylalanine (Phe) and tyrosine (Tyr) than control plants, respectively (Table 1).

**Table 1:** Amino acid profiling analysis of control and CGS‐RNAi transgenic switchgrass plants

Amino acids	Fold change (CGSRi‐1/Ctrl)	*P*‐value	Fold change (CGSRi‐3/Ctrl)	*P*‐value
**Phenylalanine**	**3.19**	**7.7E‐07**	**2.58**	**1.1E‐05**
**Tryptophan**	**3.51**	**8.6E‐05**	**3.10**	**0.00034**
Creatinine	0.67	0.01790	0.81	0.16473
**Isoleucine**	**1.62**	**0.00678**	**1.71**	**0.00291**
**Methionine**	**1.79**	**0.11575**	**1.94**	**0.06194**
**Tyrosine**	**2.66**	**9.2E‐06**	**2.27**	**8.0E‐05**
Valine	2.56	6.9E‐05	2.20	0.00047
Proline	2.24	0.00016	2.09	0.00045
Leucine	1.20	0.52888	1.21	0.52120
Hydroxyproline	6.18	0.03141	7.64	0.01203
**Threonine**	**8.53**	**0.00154**	**8.44**	**0.00167**
Alanine	4.62	2.5E‐05	3.36	0.00065
Creatine	0.53	0.00594	0.54	0.00722
Glutamine	5.92	0.00082	5.28	0.00210
Glutamate	1.30	0.44510	2.17	0.00267
Glycine	8.18	0.00015	6.16	0.00159
Serine	6.99	5.2E‐07	5.00	1.5E‐05
**Asparagine**	**51.38**	**3.9E‐08**	**54.48**	**3.5E‐08**
Citrulline	3.39	0.35298	7.09	0.01396
**Aspartate**	**3.34**	**0.01402**	**5.34**	**0.00025**
Histidine	3.69	8.4E‐05	3.62	0.00010
Arginine	3.68	8.7E‐06	3.96	3.8E‐06
**Lysine**	**8.90**	**0.01069**	**10.18**	**0.00441**
Ornithine	1.38	0.86377	3.23	0.04223
Putrescine	1.75	0.03909	2.20	0.00300
Spermidine	1.12	0.62225	1.07	0.82758

Switchgrass stems at E4 stage were collected for profiling analysis of amino acids using LC‐MS/MS. The control plants (Ctrl) were produced with pANIC8B empty vector from the same batch of experiment. Three technical replicates and two biological replicates were conducted for amino acid profiling. The mean values were used for statistical analyses. Aspartate‐derived and aromatic amino acids are shown in bold font.

### Transcriptome analysis of CGS‐RNAi transgenic switchgrass plants

To investigate the global impacts of down‐regulation of *PvCGS* on free amino acid accumulation, lignin biosynthesis and plant growth, representative lines CGSRi‐1 and CGSRi‐3 were selected for RNA‐seq analysis. Compared with control plants, a total of 847 transcripts, many of which are involved in the process of carbohydrate metabolism, the formation of precursor metabolites, and the generation of energy, were differentially expressed in transgenic lines (Figure S3). Transcript abundance of 14 genes responsible for amino acid biosynthesis was remarkably changed in transgenic lines (Table S3). Among them, the most interesting ones are those involved in the biosynthesis of Asp‐derived amino acids. Closer inspection of the data revealed a fourfold increase in expression levels of *asparagine synthetase* (*ASNS*) in the transgenic lines (Table S3). In addition, transcript abundances of *glutamate synthase* (*GOGAT*), *glutamate decarboxylase* (*GDH*) and *amidophosphoribosyltransferase* (*GPAT*) were found to be threefold to sixfold reduced (Table S3).

Previous studies have suggested that CGS is a committed enzyme controlling Met biosynthesis, which could affect lignin biosynthesis by regulating SAM and SAH levels in plants (Hanson and Roje, 2001; Kim and Leustek, 2000). Thus, we examined expression levels of genes involved in Met cycling and lignin biosynthetic pathway in CGS‐RNAi transgenic switchgrass lines. Our results showed that the transcript abundance of Met‐ and lignin‐related genes, except eight class III *peroxidases* with fourfold to 10‐fold increase, was not altered in transgenic lines compared with control plants (Table S3).

As down‐regulation of *PvCGS* caused an apparent growth stunting, we next studied expression levels of genes involved in hormone biosynthesis and signal transduction in transgenic switchgrass lines. A total of 14 hormone‐related genes were differentially altered in the transgenic lines. Among them, expression of transcripts with homology to *GA1*,* IAA6‐like*,* ARF*,* B‐ARR*,* ABF* and *ACO* was dramatically down‐regulated (Table S3).

### Down‐regulation of *PvCGS* increased both G and S lignin contents

To study the impact of down‐regulation of *PvCGS* on lignin accumulation, we first employed phloroglucinol–HCl staining to indicate the variation in lignin content in transgenic switchgrass plants. In the control internode (I2), an apparent red coloration was specially exhibited in the well‐lignified tissues such as fibres, sclerenchymas and vascular bundles (Figure 3c). In contrast, the staining intensity was substantially increased in those of transgenic plants (Figure 3c). The structure of vascular bundles in transgenic plants, however, resembled that of control plants (Figure 3c). To further confirm the enhanced lignin biosynthesis, lignin composition of stems of transgenic and control plants was detected by the thioacidolysis method. Lignin composition analysis revealed a 14% to 45% increase in G lignin monomer yield in transgenic lines (Figure 3d). Meanwhile, S lignin monomer yield was elevated by 18%–26% in the transgenic lines as well (Figure 3d).

### SAH competitively inhibited enzymatic activities of CCoAOMT and COMT

**Figure 4:**
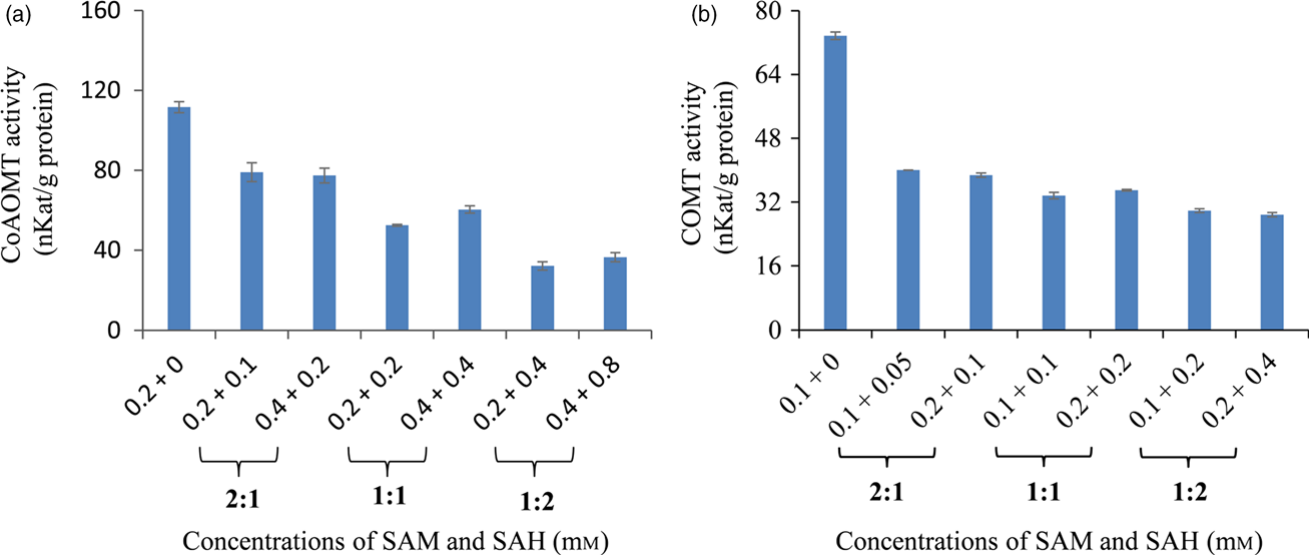
The inhibition of SAH on caffeoyl CoA *O*‐methyltransferase (CCoAOMT) and caffeic acid *O*‐methyltransferase (COMT) enzyme activities. Crude enzyme extracts were prepared from the stems of wild‐type switchgrass plants at the E4 stage, and the extractable CCoAOMT (a) and COMT (b) enzyme activities were assayed with 10 μm caffeoyl CoA and 10 μm caffeic acid, respectively, at different SAM and SAH concentrations. Values are means ± SE (*n* = 3).

Previous studies have suggested that SAH is a competitive inhibitor of methyltransferases in animal and microbe cells (Keating *et al*., 1991; Nguyen *et al*., 2001). To explore the impact of SAH alteration on lignin biosynthesis, *in vitro* enzymatic activities of CCoAOMT and COMT in crude plant extracts from wild‐type switchgrass plants were measured with various ratios of SAM/SAH. Our results revealed that both CCoAOMT and COMT activities were significantly inhibited by SAH. Moreover, the ratios of SAM/SAH rather than SAM concentrations had stronger effects on enzymatic activities of CCoAOMT and COMT (Figure 4).

### Down‐regulation of *PvSAHH1* increased SAH levels, reduced lignin accumulation and increased cell wall digestibility

**Figure 5:**
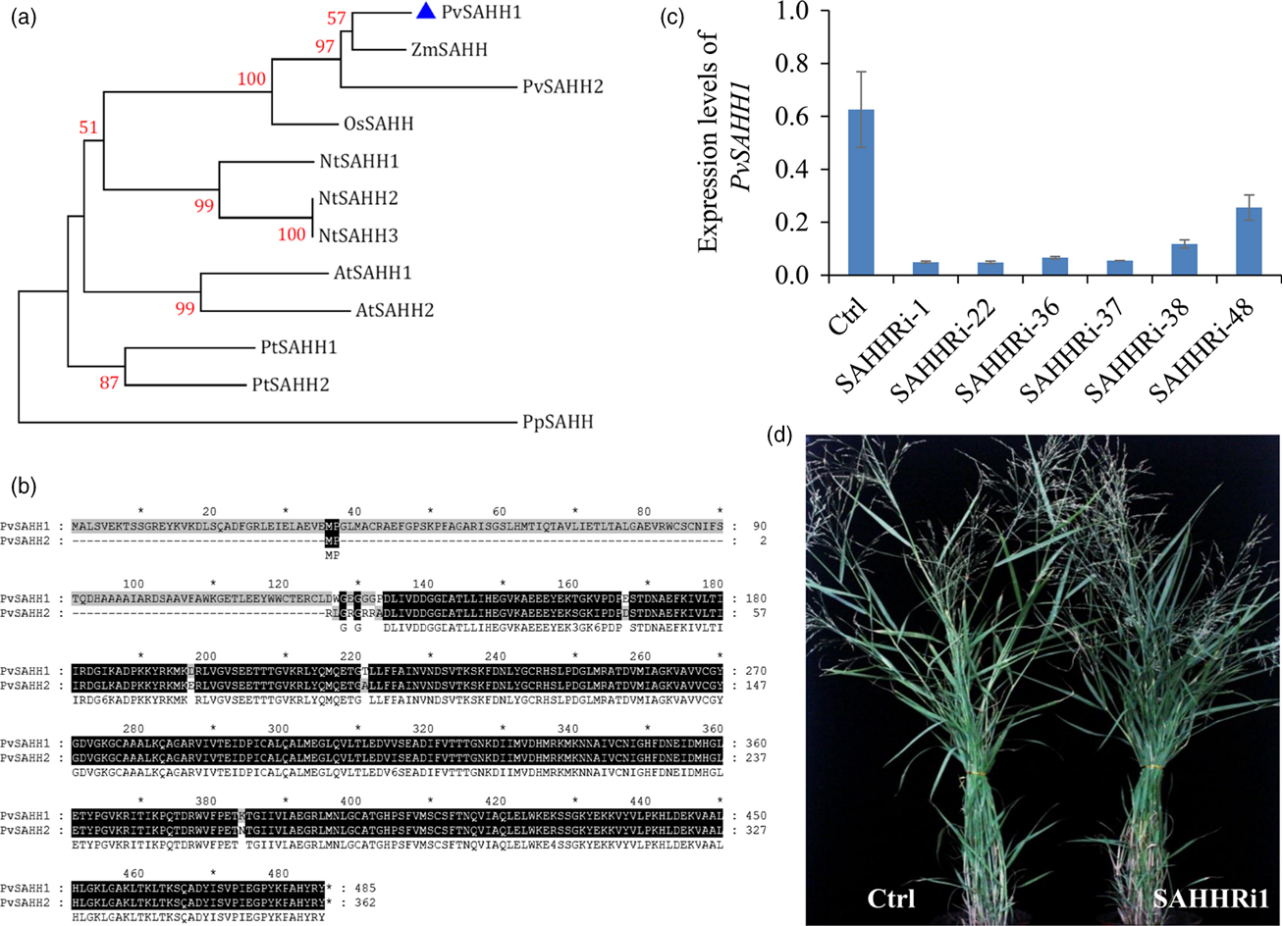
Characterization of SAHH‐RNAi transgenic switchgrass plants. (a) Phylogenetic tree of the SAHH orthologs in monocot and dicot plant species. A maximum likelihood tree was constructed in PhyML version 3.0 on the basis of multiple alignments of the deduce protein sequences from *Panicum virgatum* (PvSAHH1, Pavir.1NG430900; PvSAHH2, Pavir.6KG019200), Z*ea mays* (ZmSAHH, GRMZM2G015295), *Oryza sativa* (OsSAHH, LOC_Os11g26850), *Arabidopsis thaliana* (AtSAHH1, AT4G13940.1; AtSAHH2, AT3G23810.1), *Nicotiana tobacum* (NtSAHH1, mRNA_62654_cds; NtSAHH2, mRNA_112781_cds; NtSAHH3, mRNA_49476_cds), *Populus trichocarpa* (PtSAHH1, POPTR_0017s08610.1; PtSAHH2, POPTR_0001s32780.1) and *Physcomitrella patens* (Pp1s20_229V6.1). Bootstrap values (>50%) based on 1000 replications are indicated at nodes. The above sequence data are retrieved from Phytozome and Sol Genomics Network. (b) Sequence alignment between *PvSAHH1* and *PvSAHH2* identified from switchgrass genome database. (c) Quantitative real‐time PCR analysis of *PvSAHH1* transcript abundance in the SAHH‐RNAi transgenic lines. Switchgrass *UBQ* was used as the reference for normalization. Stems at E4 stage were collected. Value are mean ± SE (*n* = 3). (d) Morphological characterization of transgenic switchgrass plants with down‐regulation of *PvSAHH1*. The control plants (Ctrl) for SAHH‐RNAi transgenic lines were generated with pANIC8B empty vector.

To further address the role of SAH in lignin biosynthesis, we manipulated SAH contents by down‐regulation of *PvSAHH1* in switchgrass. Firstly, we cloned *PvSAHH1* (*Pavir.1NG430900.1*) and *PvSAHH2* (*Pavir.6KG019200.1*) from switchgrass, which share 96% similarity in amino acids. The phylogenetic relationship analysis shows that *PvSAHH1* and *PvSAHH2* are closely related to their orthologs in maize and rice (Figure 5a). However, *PvSAHH2* is not an intact gene, in which a 133‐bp deletion in the N‐termini causes a frameshift mutation (Figure 5b). Thus, we selected *PvSAHH1* for further studies. Temporal and spatial expression analysis of *PvSAHH1* revealed that *PvSAHH1* was highly expressed in well‐lignified tissues (Figure S4) and had a positive correlation with expressions of *CCoAOMT* and *COMT* in wild‐type switchgrass (Figure S5). To reduce SAH contents in switchgrass, we next produced 30 independent positive transgenic lines using RNAi technology. The control plants were generated with pANIC8B empty vector that was employed as the backbone for constructing SAHH‐RNAi vector. Among them, three transgenic lines SAHHRi‐1, 22 and 36 with strongly down‐regulated expression levels of *PvSAHH1* and normal phenotype were selected for further investigation (Figure 5c, d).

**Figure 6:**
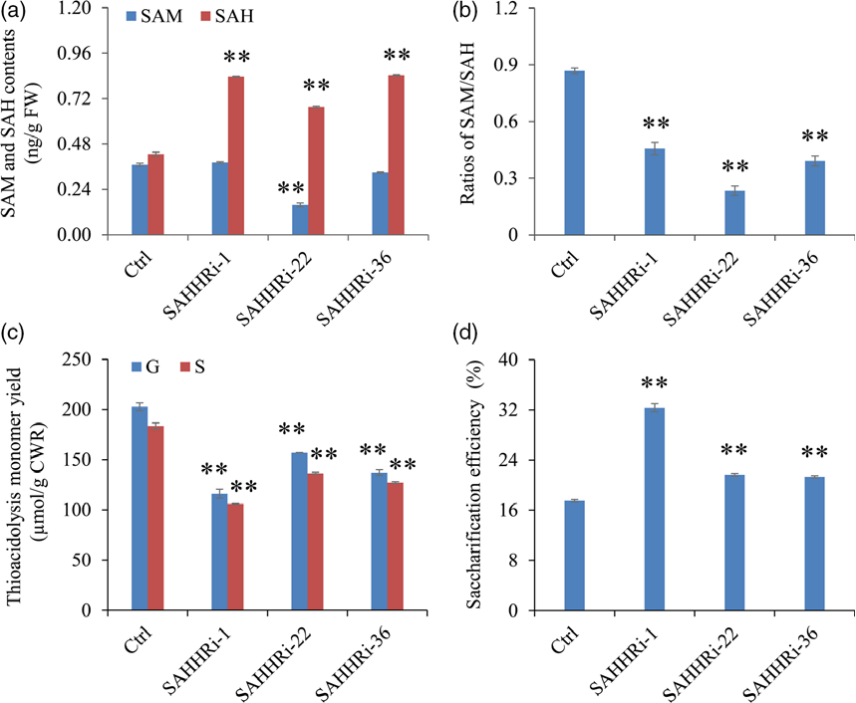
The effects of *SAHH* down‐regulation on C1 metabolites, lignin biosynthesis and cell wall digestibility of switchgrass. (a) The contents of SAM and SAH in control and transgenic switchgrass plants. (b) The ratios of SAM to SAH in control and transgenic switchgrass plants. (c) G and S lignin monomer yield of the SAHH‐RNAi transgenic lines. (d) Saccharification efficiency of cell walls of the SAHH‐RNAi transgenic lines. The control plants (Ctrl) for SAHH‐RNAi transgenic lines were generated with pANIC8B empty vector. Stems were collected from the tillers at E4 stage. FW, fresh weight; CWR, cell wall residue. Values are means ± SE (*n* = 3). One or two asterisks indicate significance corresponding to *P *<* *0.05 or 0.01 (one‐way ANOVA Dunnett's test).

Enzyme‐linked immunosorbent assay assay of intermediates in the Met cycle showed that the contents of SAH were increased by 59%–96% in transgenic switchgrass lines compared with control plants. In contrast, no consistent changes were detected in the levels of SAM and Hcy in these transgenic lines (Figure 6a, Figure S6). As a consequence, the increased SAH levels significantly reduced the ratios of SAM/SAH in all transgenic lines (Figure 6b). Furthermore, we studied the effect of elevating SAH levels on lignin biosynthesis in switchgrass. Our results showed that both G and S lignin contents were significantly reduced in the transgenic lines (Figure 6c). In addition, given the reduced lignin biosynthesis, we further assess the effect of *PvSAHH* down‐regulation on cell wall digestibility of transgenic switchgrass plants. As expected, the enzymatic hydrolysis efficiency of cell wall polysaccharides was improved by 22%–85% in the transgenic lines compared with the control plants (Figure 6d).

## Discussion

Lignocellulosic biomass mainly composed of cell walls is a sustainable source utilized as animal fodder and renewable fuels. However, the digestion of cell wall polysaccharides is not easy due to the presence of lignin (Marriott *et al*., 2016). To date, many strategies have been employed to manipulate lignin for the improvement of cell wall digestibility (Grabber *et al*., 2015; Loque *et al*., 2015). The most straightforward way is to regulate the expression levels of structural genes involved in lignin biosynthetic pathway or their transcription factors (Bonawitz and Chapple, 2010; Zhong and Ye, 2009). A system‐wide analysis of *Arabidopsis* mutants and transgenic sorghum lines with altered lignin biosynthesis has revealed that shikimate, phenylpropanoid and methyl donor pathways are closely coordinated (Scully *et al*., 2018; Vanholme *et al*., 2012). Recent studies also suggest a direct link between C1 metabolism and monolignol methylation (Byerrum *et al*., 1954; Shen *et al*., 2002; Srivastava *et al*., 2015; Tang *et al*., 2014). The regulatory mechanism of C1 metabolites on lignin biosynthesis, however, is still elusive. We show here that altering SAH contents by down‐regulating *PvCGS* or *PvSAHH1* in switchgrass can affect the biosynthesis of both G and S lignins. This information provided allows identification of more potential targets for the use in lignin bioengineering through altering SAH production.

Cystathionine γ*‐*synthase is a rate‐limiting enzyme in Met biosynthesis. Compared with dicot species, the function of CGS has yet to be investigated in monocot species. The overexpression of *CGS* in *Arabidopsis*, tobacco, alfalfa, soybean, azuki bean and potato has no effect on plant growth and development (Avraham *et al*., 2005; Hacham *et al*., 2008; Hanafy *et al*., 2013; Kreft *et al*., 2003). In contrast, down‐regulation of *CGS* in *Arabidopsis* resulted in severe growth retardation and delayed flowering (Gakière *et al*., 2000; Kim and Leustek, 2000). A similar phenotype has been observed in CGS‐RNAi transgenic switchgrass plants. In addition, cystathionine β‐lyase (CBL) is a downstream enzyme that catalyses cystathionine to form Hcy in plants (Galili *et al*., 2016). The CBL‐antisense transgenic potato plants exhibit short and bushy growth habit, small leaves and tubers and delayed flowering (Maimann *et al*., 2000). These morphological characterizations are consistent with those of CGS‐down‐regulated transgenic plants, suggesting that any attempt to dramatically impair Met biosynthesis in plants may induce the risk for abnormal growth and development. Furthermore, our transcriptome analysis revealed that down‐regulation of *CGS* in switchgrass had significant effects on biosynthesis and signal transduction pathways of hormones such as GA, auxin and ethylene. In contrast, the expression levels of lignin pathway and Met cycling genes were not changed, which excluded the impact of SAM‐mediated DNA methylation on their expression. Taken together, these results suggest that manipulation of Met metabolism can trigger a complicated regulatory network in plants.

Previous studies have shown that the overexpression of *CGS* in *Arabidopsis* elevates Met contents dramatically (Gakière *et al*., 2002; Kim *et al*., 2002). However, down‐regulation of *CGS* in *Arabidopsis* changes the levels of free Met slightly (Gakière *et al*., 2000; Kim and Leustek, 2000). Consistently, down‐regulation of *CGS* in switchgrass had little effect on Met accumulation. These results suggest that other pathways such as Met salvage, SMM‐mediated transportation and protein degradation may compensate for the loss of Met in plants (Cohen *et al*., 2017b; Kreft *et al*., 2003). Given the fact that Met is synthesized from Asp, we further examined the levels of Asp‐derived amino acids. Our results revealed a significant increase in these Asp family members. Among them, the levels of Thr were elevated by 8.5‐fold in the transgenic lines compared with the control plants. It is consistent with the previous observation in *Arabidopsis* in which suppression of *CGS* led to threefold to fivefold increase in the contents of free Thr (Kim and Leustek, 2000). TS competes with CGS for the common substrate *O*‐phosphohomoserine to shunt metabolic flux into Thr biosynthesis. Little change in the expression levels of *TS* was observed in the CGS‐RNAi transgenic switchgrass lines, suggesting that the increased Thr levels were mainly caused by the elevated substrate concentrations.

The effect of CGS on lignin biosynthesis was further studied in switchgrass. Our results show that the expression levels of *PvCGS* are raised during the process of internode lignification, consistent with the observation of *PvSAHH1* in switchgrass (Figure S4) and *FPGS*,* MTHFR* and *SAMS3* in *Arabidopsis* and maize (Shen *et al*., 2002; Srivastava *et al*., 2015; Tang *et al*., 2014). Down‐regulation of *CGS* in switchgrass increased the contents of SAH consistently. However, the levels of SAM and Hcy were varied among the transgenic lines, suggesting that the cells temperately adjusted their biosynthetic machinery to maintain the Met levels by controlling other metabolic flux towards SAM and Hcy. Nevertheless, all transgenic switchgrass lines still exhibited the elevated ratios of SAM/SAH. Furthermore, a remarkable inhibition of SAH on both CCoAOMT and COMT activities was observed *in vitro*. Thus, we suspect that the reduced SAH level was one of the reasons that can improve methylation efficiency of monolignols. To further test this assumption, we produced SAHH‐RNAi transgenic switchgrass lines in which SAH levels were significantly increased and the ratios of SAM/SAH were dramatically decreased. As expected, the contents of G and S lignins were reduced and, as a consequence, cell wall saccharification efficiency was improved in the transgenic switchgrass plants. Thus, our results suggest that maintenance of appropriate SAH levels is crucial for lignin biosynthesis in switchgrass.

It is notable that a sufficient substrate supply is another key factor for high product yield in monolignol methylations apart from the enhanced methyltransferase enzymatic activity. Moreover, previous research has suggested that the overexpression of *COMT* cannot increase lignin accumulation in *Arabidopsis* (Goujon *et al*., 2003). Therefore, we attempt to seek whether other possibilities could enhance lignin biosynthesis in CGS‐RNAi transgenic switchgrass plants. Amino acid profiling analysis revealed a significant increase in the contents of Phe and Tyr. Recent study indicates that both Phe and Tyr catalysed by PAL and TAL in grass species can provide precursors for lignin biosynthesis (Barros *et al*., 2016). Thus, the elevated levels of Phe and Tyr as well as the reduced SAH inhibition for CCoAOMT and COMT contribute to the significant increase in G and S lignins in CGS‐RNAi transgenic switchgrass plants. The mechanism inducing the accumulation of Phe and Tyr in the CGS‐down‐regulated background remains largely unknown, but it deserves future investigation.

In summary, we studied the function of *CGS* for the first time in monocot species. Our results showed that down‐regulation of *CGS* in switchgrass enhanced lignin biosynthesis through elevating Phe and Tyr contents and reducing SAH levels. Moreover, SAH, as a strong inhibitor of monolignol methylation reactions, plays an important role in lignin biosynthesis. Genetic manipulation of SAH can impair both G and S lignin biosynthesis and therefore improve cell wall digestibility. Thus, our work suggests that genes involved in Met metabolism are new valuable targets for cell wall bioengineering.

## Experimental procedures

### Plant materials and growth conditions

A lowland‐type of switchgrass cultivar, Alamo (2*n* = 4 × =36), was employed for genetic transformation and lignin modification. Switchgrass plants were grown in the glasshouse at 26 °C with 16‐h light (390 μmol/m^2^/s). The development stages of switchgrass in our glasshouse were identified following the criteria described by Moore *et al*., 1991.

### Identification and cloning of *PvCGS* and *PvSAHH1*

*Arabidopsis thaliana* nucleic acid sequences *AtCGS* (AT3G01120) and *AtSAHH1* (AT4G13940) were used as a query to BLAST against the *Panicum virgatum* v4.1 genome sequences (Phytozome). *PvCGS* (Pavir.9NG556700) and *PvSAHH1* (Pavir.1NG430900) were identified as the most homologous genes. The predicted cDNA sequences of *PvCGS* and *PvSAHH1* were used to design primers for cloning the open‐reading frames of *PvCGS* and *PvSAHH1*. Alignment of multiple sequences and phylogenetic tree analysis of *PvCGS*,* PvSAHH1* and their orthologs in six genome‐sequenced species (*Panicum virgatum*,* Zea mays*,* Oryza sativa*,* Arabidopsis thaliana*,* Nicotiana tabacum* and *Populus trchocarpa*) were conducted using MEGA 5 software suite.

### Gene expression pattern analysis

Internodes, leaf sheaths and leaf blades of internode 2 (I2), internode 3 (I3) and internode 4 (I4) at the E4 stage were collected, and the I2 internodes at E2, E3, E4 and R1 stages were prepared, respectively. All tissue samples were immediately frozen in liquid nitrogen and stored at −80 °C. Total RNA was isolated using the TRIZOL reagent according to the manufacturer's supplied protocol (Thermo‐Fisher Scientific) and subjected to reverse transcription with Superscript PrimeScript™RT reagent Kit (TaKaRa, Japan) after treatment with Turbo DNase I (TaKaRa, Japan). Quantitative reverse transcription–polymerase chain reaction (qRT–PCR) was performed to analyse the transcript abundance of *PvCGS* and *PvSAHH1* in switchgrass (Fu *et al*., 2012). The data were normalized against the reference genes of *PvUBQ* (GenBank accession no: HM209468). Primer pairs used for qRT–PCR are listed in Table S4.

### Generation of transgenic switchgrass plants

A 457‐bp and 480‐bp cDNA fragments located in the conserved regions of *PvCGS* and *PvSAHH1*, respectively, were cloned into pANIC8B, an RNAi binary vector (Mann *et al*., 2012). The primers used in gene cloning and RNAi vector construction were listed in Table S4. The verified recombinant constructs of pANIC8B‐CGSRi and pANIC8B‐SAHHRi were transferred into *Agrobacterium tumefaciens* strain *AGL1*.

A highly embryogenic callus line with single genotype generated by screening large scale of switchgrass Alamo seed‐derived calli were used for *Agrobacterium*‐mediated transformation following the procedure described by Xi *et al*. (2009). The independent positive transgenic lines were subjected to transcript abundance of endogenous *PvCGS* and *PvSAHH1* by qRT–PCR.

### Characterization of plant growth and development

For morphological characterization, plant height and flowering time were measured using 6‐month‐old transgenic plants after cutting (Fu *et al*., 2012). The I2 internodes were used for measuring the internode length, internode diameter, leaf sheath length and leaf blade length and width.

### RNA‐seq analysis of transgenic switchgrass plants

Stems at the E4 stage were collected from control and CGS‐RNAi transgenic switchgrass plants. Total RNA was extracted for RNA library preparation and deep sequencing. The genome sequences and annotated data of switchgrass were downloaded from phytozome. We aligned all reads from each sample to the reference genome of *P. virgatum* (v4.1) using tophat v2.1.0, allowing up to five mismatches (Trapnell *et al*., 2009). The expression levels of genes were measured using Cufflink v2.1.1 (Trapnell *et al*., 2012), and the differential expression analysis between control and transgenic switchgrass plants was calculated using DEseq (Anders and Huber, 2010). The volcano plot and heatmap of differentially expressed genes were created using R package (Lawrence, 2016).

### Amino acid profiling of transgenic switchgrass plants

Stems at the E4 stage were collected and homogenized in liquid nitrogen. For amino acid profiling analysis, 500 mg of powdered tissue was extracted and derivatized with diethyl ethoxymethylenemalonate as described by Cai *et al*. (2017). The aminoenone derivatives were identified and quantified by liquid chromatography coupled to tandem mass spectrometry (LC‐MS/MS). The Agilent 1290 Infinity LC equipped with a Zic‐HILIC column (3.5 μm, 2.1 mm × 150 mm) was employed for a high‐resolution separation of amino acids. The injection volume was 2 μL. The mobile phase consisted of eluent A (25 mm HCOONH4 with 0.08% HCOOH) and eluent B (acetonitrile with 0.1% HCOOH), and separation was achieved using a linear gradient of B eluent (0–12 min, 90% B to 70% B; 12–18 min, 70% B to 50% B; 18–25 min, 50% B to 40% B; 25–30 min, 40% B) at a flow rate of 250 μL/min. The column was maintained at 40 °C. All the mass spectra were acquired using a mass selective detector (5500 QTRAP) coupled with an electrospray ionization (ESI) source. Mass spectra from positive‐ion ESI were recorded over the range 50–2200 m/z. Boxplots of amino acids were generated using R package (Lawrence, 2016).

### Measurement of SAM, SAH and Hcy by enzyme‐linked immunosorbent assay (ELISA)

To detect the contents of SAM, SAH and Hcy, the stems of control and transgenic switchgrass plants at the E4 stage were collected. Approximately 50 mg fresh biomass was ground in phosphate buffered saline (0.01 m, pH = 7.2). After centrifuging at 2348 ***g*** for 15 min, the extracts were immediately used to measure the contents of SAM, SAH and Hcy according to the protocol of ELISA kit (Shanghai Enzyme‐linked Biotechnology, China).

### CCoAOMT and COMT enzyme activity assay

The stems of wild‐type switchgrass plants were collected at the E4 stage and homogenized in liquid nitrogen. Powdered tissue (about 500 mg) was extracted for 3 h at 4 °C in extraction buffer (Liu *et al*., 2012) and then centrifuged at 17 900 ***g*** for 20 min at 4 °C. COMT and CCoAOMT activities in crude plant extracts were detected as described by Liu *et al*. (2012). The inhibition effect of SAH on CCoAOMT and COMT activities were determined in reactions with different ratios of SAM to SAH including 2:1, 1:1 and 1:2.

### Histochemical assay

The I2 internodes were collected from the stems at the E4 stage for histochemical assay. The internode cross sections were stained with phloroglucinol–HCl reagent for lignin characterization as described previously (Chen *et al*., 2002). The micrographs were taken under a Nikon Microphot‐FX system with a Nikon DXM 1200 colour camera (Nikon, Japan).

### Thioacidolytic analysis of residual lignins

The stems were harvested at the E4 stage. The collected samples were ground in liquid nitrogen and lyophilized. Lyophilized extractive‐free material was used for lignin analysis. The thioacidolysis method was used to detect lignin composition (Lapierre *et al*., 1995). G and S lignins were identified and quantified by gas chromatography–mass spectrometry (GC‐MS) using a Hewlett‐Packard 5890 series II gas chromatograph with a 5971 series mass selective detector (column HP‐1, 60 m × 0.25 mm, film thickness 0.25 μm).

### Detection of saccharification efficiency

Saccharification efficiency of switchgrass cell walls was detected as described by Fu *et al*. (2011). The amount of fermentable sugars was measured by the phenol–sulphuric acid assay method (Dubois *et al*., 1956). Saccharification efficiency was determined as the ratio of sugars released by enzymatic hydrolysis to the amount of total sugars present in cell wall materials before enzymatic hydrolysis treatment.

### Statistical analysis

Samples were collected from three biological replicates of each transgenic line and control plant except profiling analysis of amino acids. Three technical replicates and two biological replicates were conducted for amino acid profiling. The mean values were used for statistical analyses. Data from each trait were subjected to one‐way ANOVA. The significance of treatments was tested at the *p *=* *0.05 level. Standard errors were provided in all tables and figures as appropriate.

## Conflict of interest

The authors declare no conflict of interest.

## Supporting information


**Figure S1** Alignment of PvCGS and AtCGS amino acid sequences.
**Figure S2** The contents of homocysteine (Hcy) in control and CGS‐RNAi transgenic switchgrass plants.
**Figure S3** Transcriptome analysis of CGS‐RNAi transgenic switchgrass plants by RNA‐seq.
**Figure S4** Quantitative RT–PCR analysis of *PvSAHH1* expression levels in different tissues.
**Figure S5** Correlationships between expression levels of *PvSAHH1* and *PvCOMT*/*PvCCoAMT*.
**Figure S6** The contents of homocysteine (Hcy) in control and SAHH‐RNAi transgenic switchgrass plants.
**Table S1** The temporal and spatial expression of *PvCGS*,* PvSAMS*, and *PvSAHH* in wild type switchgrass plants.
**Table S2** Morphological characterization of CGS‐RNAi transgenic switchgrass plants.
**Table S3** Genes differentially expressed in CGS‐RNAi transgenic switchgrass plants.
**Table S4** Primers used in this study.
