# Supplementary material for: Alteration of S‐adenosylhomocysteine levels affects lignin biosynthesis in switchgrass
Source: Plant Biotechnol J. 2018 Jun 6;16(12):2016–26. doi: 10.1111/pbi.12935 (PMC6230947; doi:10.1111/pbi.12935)
Supplement: Supplementary file 1 — Figure S1 Alignment of PvCGS and AtCGS amino acid sequences. Figure S2 The contents of homocysteine (Hcy) in control and CGS‐RNAi transgenic switchgrass plants. Figure S3 Transcriptome analysis of CGS‐RNAi transgenic switchgrass plants by RNA‐seq. Figure S4 Quantitative RT–PCR analysis of PvSAHH1 expression levels in different tissues. Figure S5 Correlationships between expression levels of PvSAHH1 and PvCOMT/PvCCoAMT. Figure S6 The contents of homocysteine (Hcy) in control and SAHH‐RNAi transgenic switchgrass plants. Table S1 The temporal and spatial expression of PvCGS, PvSAMS, and PvSAHH in wild type switchgrass plants. Table S2 Morphological characterization of CGS‐RNAi transgenic switchgrass plants. Table S3 Genes differentially expressed in CGS‐RNAi transgenic switchgrass plants. Table S4 Primers used in this study. [file PBI-16-2016-s001.doc]

**Supporting Information**

**Figure S1.** Alignment of PvCGS and AtCGS amino acid sequences.

**Figure S2.** The contents of homocysteine (Hcy) in control and CGS-RNAi transgenic switchgrass plants. Stems were collected from the tillers at E4 stage. The levels of Hcy were determined by ELISA. Values are means ± SE (n=3).

**Figure S3.** Transcriptome analysis of CGS-RNAi transgenic switchgrass plants by RNA-seq.

(a) Volcano plot showed the different expressed genes in CGS-RNAi transgenic switchgrass plants compared with control plants. The red and green dots represent the up- and down-regulated genes with *p* < 0.05, respectively. (b) Gene ontology (GO) enrichment analysis of differentially expressed genes between control and CGS-RNAi transgenic plants.

**Figure S4.** Quantitative RT-PCR analysis of *PvSAHH1* expression levels in different tissues.

E2, Elongation 2 stage; E3, Elongation 3 stage; E4, Elongation 4 stage; R1, Reproduction 1 stage; I2, Internode 2; I3, Internode 3; I4, Internode 4. Switchgrass *UBQ* was used as the reference for normalization. Value are mean ± SE (n=3).

**Figure S5.** Correlationships between expression levels of *PvSAHH1* and *PvCOMT*/*PvCCoAMT*.

The data was downloaded from Switchgrass Functional Genomics Server (https://switchgrassgenomics.noble.org). The sample points includes root, crown, node, leaf blade, leaf sheath, middle 1/5 fragment of internode 3, vascular bundle isolated from 1/5 fragment of internode 3, top 1/5 fragment of internode 4, middle 1/5 fragment of internode 4, bottom 1/5 fragment of internode 4 at the E4 stage, inflorescence of rachis, primary and secondary branch meristem initiation stages (0.5-3.0 mm), inflorescence of glume and floret development stages (10-20 mm), inflorescence (50-150 mm), and inflorescence (>200 mm). The correlations between the expression levels of *PvSAHH1* and *PvCOMT*/*PvCCoAOMT* were statistically significant (*p* < 0.05).

**Figure S6.** The contents of homocysteine (Hcy) in control and SAHH-RNAi transgenic switchgrass plants.

Stems were collected from the tillers at E4 stage. The levels of Hcy were determined by ELISA. Values are means ± SE (n=3).

**Table S1. The temporal and spatial expression expression of *PvCGS*, *PvSAMS*, and *PvSAHH* in wild type switchgrass plants.**

| Tissues at different development stages | *PvCGS* | *PvSAHH* | *PvSAMS* |
| --- | --- | --- | --- |
| I2 at the E2 stage | 0.0197±0.0025 | 0.2966±0.0378 | 0.2052±0.0021 |
| I2 at the E3 stage | 0.0772±0.0008 | 0.6575±0.0068 | 0.2588±0.0018 |
| I2 at the E4 stage | 0.0651±0.0043 | 0.5037±0.0140 | 0.1708±0.0012 |
| I2 at the R1 stage | 0.1009±0.0035 | 0.5529±0.0057 | 0.2261±0.0008 |
| I4 at the E4 stage | 0.0355±0.0046 | 0.5587±0.0077 | 0.1844±0.0064 |
| I3 at the E4 stage | 0.0633±0.0037 | 0.3789±0.0026 | 0.1405±0.0155 |
| Leaf sheath 4 at the E4 stage | 0.0353±0.0018 | 0.1212±0.0101 | 0.0392±0.0050 |
| Leaf sheath 3 at the E4 stage | 0.0434±0.0005 | 0.0945±0.0091 | 0.0694±0.0014 |
| Leaf sheath 2 at the E4 stage | 0.0493±0.0032 | 0.1387±0.0010 | 0.0656±0.0023 |
| Leaf blade 4 at the E4 stage | 0.0223±0.0004 | 0.0390±0.0014 | 0.0225±0.0004 |
| Leaf blade 3 at the E4 stage | 0.0466±0.0021 | 0.0405±0.0053 | 0.0201±0.0003 |
| Leaf blade 3 at the E4 stage | 0.0125±0.0013 | 0.0866±0.0006 | 0.0289±0.0005 |

* The tillers at three elongation stages (E2, E3, and E4) and one reproductive stage (R1) are associated with a significantly progressive lignification of cell walls. Therefore, we collected the second internode (I2) from the corresponding tillers at the above stages, respectively. The successive internodes (I2-4) and their corresponding leaf sheaths and leaf blades were dissected from the tillers harvested at the E4 stage. The expression levels of *PvCGS*, *PvSAHH*, and *PvSAMS* were measured by quantitative real-time PCR. Switchgrass *UBQ* was used as the reference for normalization.

**Table S2. Morphological characterization of CGS-RNAi transgenic switchgrass plants.**

|  | Plant height  (cm) | Leaf blade length (cm) | Leaf blade width (cm) | Leaf sheath length(cm) | Internode  length (mm) | Internode  diameter (mm) | Range of  internode number | Flowering time(day) |
| --- | --- | --- | --- | --- | --- | --- | --- | --- |
| Ctrl | 130.81±2.16 | 50.92±1.08 | 1.65±0.49 | 17.98±0.09 | 21.61±0.45 | 3.04±0.10 | 5-6 | 92±2 |
| CGSRi-1 | 50.04±0.89^**^ | 28.64±0.70^**^ | 1.75±0.35 | 6.92±0.17^**^ | 9.38±0.11^**^ | 3.86±0.15 | 5-6 | >360 |
| CGSRi-2 | 48.72±0.69^**^ | 27.41±0.71^**^ | 1.59±0.20 | 7.06±0.20^**^ | 9.22±0.12^**^ | 3.86±0.13 | 5-6 | >360 |
| CGSRi-3 | 49.15±0.45^**^ | 29.51±0.59^**^ | 1.42±0.20 | 7.00±0.15^**^ | 9.32±0.10^**^ | 4.02±0.06 | 5-6 | >360 |

*Plant height of switchgrass was measured after 6-month growth in the greenhouse. The 6-month-old tillers were used to measure internode length (internode 2), internode diameter (internode 2), internode number, leaf blade length and width. Five tillers were measured for each replicate. A value of 5-6 means there are, on average, 5-6 visible internodes for a given line at the time of harvest. Control plants (Ctrl) were produced with pANIC8B empty vector from the same batch of experiment. Values are mean ± SE (n=3). Asterisk indicates significance corresponding to *p* < 0.05 (One way ANOVA, Dunnett’s test).

**Table S3.** Genes differentially expressed in CGS-RNAi transgenic switchgrass plants.

Sheet 1

| **Gene_id** | **log2FoldChange** | **q-value** | **GeneDescription** |
| --- | --- | --- | --- |
| Novel00811 | -10.043 | 1.3E-25 | - |
| Pavir.5NG364900.v4.1 | -9.784 | 2.4E-36 |  |
| Pavir.5NG484100.v4.1 | -9.4976 | 3.77E-38 |  |
| Pavir.3NG182300.v4.1 | -8.9163 | 5.22E-18 | beta glucosidase 42 |
| Pavir.J578600.v4.1 | -8.6028 | 0.001068 |  |
| Pavir.7KG064900.v4.1 | -8.45 | 2.52E-06 | basic helix-loop-helix (bHLH) DNA-binding superfamily protein |
| Pavir.5NG540400.v4.1 | -7.9844 | 3.32E-10 |  |
| Pavir.5NG522400.v4.1 | -7.7702 | 2.01E-13 | photosystem II reaction center W |
| Pavir.5NG517300.v4.1 | -7.6139 | 6.22E-10 | Subtilase family protein |
| Novel00675 | -7.5809 | 6.07E-10 | - |
| Pavir.5KG724200.v4.1 | -7.3883 | 0.000937 | Bifunctional inhibitor/lipid-transfer protein/seed storage 2S albumin superfamily protein |
| Pavir.5NG480200.v4.1 | -7.3517 | 1.03E-28 | BURP domain-containing protein |
| Pavir.7KG208900.v4.1 | -7.3052 | 2.39E-13 | Regulator of chromosome condensation (RCC1) family protein |
| Pavir.5NG399000.v4.1 | -7.305 | 4.76E-16 | Rab5-interacting family protein |
| Pavir.3KG477400.v4.1 | -7.1282 | 1.36E-07 |  |
| Pavir.7KG190800.v4.1 | -7.0052 | 0.006371 | Transmembrane amino acid transporter family protein |
| Pavir.6NG136900.v4.1 | -6.9377 | 2.88E-18 | Regulator of chromosome condensation (RCC1) family protein |
| Pavir.7KG252000.v4.1 | -6.885 | 1.51E-06 | beta-glucosidase 45 |
| Pavir.5NG620300.v4.1 | -6.7608 | 4.92E-06 |  |
| Pavir.7KG141500.v4.1 | -6.5015 | 0.006369 | UDP-glucosyl transferase 85A2 |
| Pavir.7KG094300.v4.1 | -6.0851 | 0.000929 |  |
| Pavir.6KG304700.v4.1 | -6.0809 | 0.000937 |  |
| Pavir.5NG318500.v4.1 | -6.0246 | 0.005141 | alpha/beta-Hydrolases superfamily protein |
| Pavir.3NG077600.v4.1 | -5.9927 | 0.001351 |  |
| Pavir.5NG417100.v4.1 | -5.9788 | 0.002293 | Werner syndrome-like exonuclease |
| Pavir.6NG349600.v4.1 | -5.9253 | 4.7E-06 | beta-1,2-xylosyltransferase |
| Pavir.5NG577700.v4.1 | -5.8848 | 0.003033 | Uncharacterised conserved protein UCP015417, vWA |
| Pavir.3NG152800.v4.1 | -5.849 | 0.003789 | Protein kinase family protein |
| Pavir.5NG411100.v4.1 | -5.841 | 0.023682 | G-box binding factor 3 |
| Pavir.5NG523100.v4.1 | -5.7894 | 1.49E-05 | mitochondrially targeted single-stranded DNA binding protein |
| Pavir.6NG047100.v4.1 | -5.7865 | 0.00498 | Hyaluronan / mRNA binding family |
| Pavir.5NG523200.v4.1 | -5.6517 | 2.39E-08 | Signal recognition particle, SRP54 subunit protein |
| Pavir.1KG130300.v4.1 | -5.5915 | 6.78E-11 | G-protein-coupled receptor 1 |
| Pavir.5NG548700.v4.1 | -5.4162 | 0.000252 | F-box family protein |
| Pavir.7KG108200.v4.1 | -5.3984 | 0.032324 | Major facilitator superfamily protein |
| Pavir.6NG316500.v4.1 | -5.3903 | 7.41E-10 | RNA binding (RRM/RBD/RNP motifs) family protein |
| Pavir.5KG728900.v4.1 | -5.366 | 0.000842 | alpha/beta-Hydrolases superfamily protein |
| Pavir.7KG128100.v4.1 | -5.3328 | 0.000523 | Ribosomal L29 family protein |
| Pavir.3KG067600.v4.1 | -5.3237 | 0.000516 | calcium-dependent protein kinase 32 |
| Pavir.6NG250200.v4.1 | -5.3225 | 8.1E-09 | cyclin-dependent kinase C;1 |
| Pavir.7NG030500.v4.1 | -5.292 | 7.93E-06 | F-box family protein |
| Pavir.5NG554200.v4.1 | -5.2858 | 1.14E-07 |  |
| Pavir.5NG501000.v4.1 | -5.1694 | 4.27E-06 |  |
| Pavir.5KG635300.v4.1 | -5.0728 | 1.47E-07 | Reticulon family protein |
| Novel00715 | -5.0575 | 0.000149 | - |
| Pavir.7KG194800.v4.1 | -4.9654 | 1.26E-07 | Gibberellin-regulated family protein |
| Pavir.5NG442900.v4.1 | -4.9522 | 1.01E-07 | Homeodomain-like/winged-helix DNA-binding family protein |
| Pavir.4NG092200.v4.1 | -4.8994 | 0.000468 | Duplicated homeodomain-like superfamily protein |
| Pavir.J470600.v4.1 | -4.8923 | 1.76E-13 | BEL1-like homeodomain 8 |
| Pavir.7KG020900.v4.1 | -4.7901 | 1.48E-10 | Thioredoxin superfamily protein |
| Pavir.5NG475800.v4.1 | -4.7674 | 9.23E-07 |  |
| Pavir.5NG482300.v4.1 | -4.717 | 2.01E-05 | UDP-glucosyl transferase 88A1 |
| Pavir.5NG620100.v4.1 | -4.6559 | 6.32E-07 | Cytochrome C1 family |
| Pavir.5NG262400.v4.1 | -4.6221 | 6.64E-14 |  |
| Pavir.5NG502600.v4.1 | -4.4765 | 2.26E-11 | Basic-leucine zipper (bZIP) transcription factor family protein |
| Pavir.6NG323000.v4.1 | -4.4583 | 0.007337 | Myzus persicae-induced lipase 1 |
| Pavir.5NG456200.v4.1 | -4.4214 | 3.04E-06 | Protein kinase superfamily protein |
| Pavir.5NG239100.v4.1 | -4.3213 | 0.003655 | nitrate transporter2.5 |
| Pavir.7KG192000.v4.1 | -4.3201 | 3.08E-06 | vacuolar iron transporter 1 |
| Pavir.7KG219300.v4.1 | -4.3059 | 0.003156 |  |
| Novel00912 | -4.2677 | 0.000287 | - |
| Pavir.5NG500100.v4.1 | -4.2571 | 0.019709 | transferases, transferring glycosyl groups |
| Pavir.5NG286100.v4.1 | -4.1986 | 0.024151 | NHL domain-containing protein |
| Novel00670 | -4.1838 | 0.00549 | - |
| Pavir.5NG534100.v4.1 | -4.1266 | 3.91E-09 | uclacyanin 1 |
| Pavir.6NG370300.v4.1 | -4.0935 | 0.009404 |  |
| Pavir.6NG144800.v4.1 | -4.088 | 0.041054 |  |
| Pavir.5NG406000.v4.1 | -4.0754 | 0.037802 |  |
| Pavir.8KG186200.v4.1 | -4.0628 | 0.00017 | Core-2/I-branching beta-1,6-N-acetylglucosaminyltransferase family protein |
| Pavir.5NG493400.v4.1 | -4.0516 | 0.00341 | heat shock protein 101 |
| Pavir.5NG533200.v4.1 | -4.0509 | 3.5E-08 |  |
| Pavir.5NG458900.v4.1 | -4.0474 | 0.000468 |  |
| Pavir.5NG478500.v4.1 | -4.0057 | 0.001582 |  |
| Pavir.6NG046400.v4.1 | -3.9599 | 0.000134 | Tetratricopeptide repeat (TPR)-like superfamily protein |
| Pavir.1NG524800.v4.1 | -3.9531 | 0.021445 | glycosyl hydrolase 9A4 |
| Pavir.6NG333600.v4.1 | -3.9325 | 1.26E-07 |  |
| Pavir.6NG307600.v4.1 | -3.924 | 3.34E-05 | RAB GTPase homolog A5D |
| Pavir.5NG500900.v4.1 | -3.9201 | 0.001339 | Nuclear transport factor 2 (NTF2) family protein |
| Pavir.7KG222400.v4.1 | -3.8587 | 0.019737 | Protein of unknown function, DUF642 |
| Pavir.J549200.v4.1 | -3.7737 | 5.42E-05 | beta glucosidase 42 |
| Pavir.7KG233200.v4.1 | -3.7737 | 0.032375 | Plant protein of unknown function (DUF247) |
| Pavir.7KG014400.v4.1 | -3.7534 | 0.000516 | Eukaryotic aspartyl protease family protein |
| Pavir.5NG503100.v4.1 | -3.7445 | 0.01807 |  |
| Pavir.5NG271200.v4.1 | -3.741 | 7.5E-08 | dihydroflavonol 4-reductase |
| Pavir.5NG568800.v4.1 | -3.7368 | 1.04E-07 | sorting nexin 2B |
| Pavir.7KG227300.v4.1 | -3.6835 | 1.79E-06 | damaged DNA binding;DNA-directed DNA polymerases |
| Pavir.5NG522300.v4.1 | -3.6595 | 2.56E-05 | Glycosyl hydrolase family 47 protein |
| Pavir.5NG490600.v4.1 | -3.6083 | 2.62E-09 | hexokinase 1 |
| Pavir.2NG136100.v4.1 | -3.5552 | 1.9E-07 | FK506-binding protein 15 kD-1 |
| Pavir.5NG328500.v4.1 | -3.5437 | 5.59E-05 | SBP (S-ribonuclease binding protein) family protein |
| Pavir.5NG500800.v4.1 | -3.5388 | 0.000823 | Transducin/WD40 repeat-like superfamily protein |
| Pavir.5NG511400.v4.1 | -3.5298 | 7.48E-06 | uvrB/uvrC motif-containing protein |
| Pavir.2NG123300.v4.1 | -3.5236 | 0.014792 | Agenet domain-containing protein |
| Pavir.5NG486500.v4.1 | -3.4606 | 5.98E-06 | profilin 5 |
| Pavir.6KG378500.v4.1 | -3.4474 | 0.03857 | Nucleotide-diphospho-sugar transferase family protein |
| Pavir.9NG236800.v4.1 | -3.4222 | 0.007346 | Disease resistance-responsive (dirigent-like protein) family protein |
| Pavir.5NG614200.v4.1 | -3.4064 | 4.27E-06 | Protein of unknown function (DUF3754) |
| Pavir.5NG460800.v4.1 | -3.3951 | 0.014663 | Protein of unknown function, DUF599 |
| Pavir.2NG645000.v4.1 | -3.384 | 0.001009 |  |
| Pavir.5NG415200.v4.1 | -3.3662 | 1.54E-06 | AMP-dependent synthetase and ligase family protein |
| Pavir.7KG312500.v4.1 | -3.3585 | 7.93E-06 | cellulose synthase-like B4 |
| Pavir.6NG278200.v4.1 | -3.3549 | 0.016455 | basic helix-loop-helix (bHLH) DNA-binding superfamily protein |
| Pavir.6NG349500.v4.1 | -3.3437 | 0.048602 | tonoplast dicarboxylate transporter |
| Pavir.7KG054700.v4.1 | -3.3002 | 0.000273 | Flavin-binding monooxygenase family protein |
| Pavir.5NG531800.v4.1 | -3.2769 | 0.027024 | diacylglycerol kinase 5 |
| Pavir.5NG583800.v4.1 | -3.266 | 0.020275 | Pathogenesis-related thaumatin superfamily protein |
| Pavir.7KG008000.v4.1 | -3.243 | 0.002081 | NAD(P)-linked oxidoreductase superfamily protein |
| Pavir.7KG117000.v4.1 | -3.223 | 0.020263 | Domain of unknown function (DUF1767) |
| Pavir.5NG637400.v4.1 | -3.2138 | 0.000141 | ARM repeat superfamily protein |
| Pavir.5NG473400.v4.1 | -3.2091 | 7.23E-08 | NADP-malic enzyme 3 |
| Pavir.7KG283900.v4.1 | -3.1957 | 0.003789 |  |
| Pavir.7KG126700.v4.1 | -3.1923 | 0.012667 | Terpenoid cyclases/Protein prenyltransferases superfamily protein |
| Novel00720 | -3.1881 | 0.003189 | - |
| Pavir.5NG630700.v4.1 | -3.1765 | 7.69E-05 | Predicted pyridoxal phosphate-dependent enzyme, YBL036C type |
| Pavir.5NG587200.v4.1 | -3.1618 | 0.001576 | S-adenosyl-L-methionine-dependent methyltransferases superfamily protein |
| Pavir.6NG337400.v4.1 | -3.1591 | 0.029771 | NB-ARC domain-containing disease resistance protein |
| Pavir.5KG663900.v4.1 | -3.1573 | 0.019488 | Cell division control, Cdc6 |
| Pavir.5NG479800.v4.1 | -3.1184 | 0.000367 | ovate family protein 12 |
| Pavir.5NG109300.v4.1 | -3.1175 | 0.008152 | S-adenosyl-L-methionine-dependent methyltransferases superfamily protein |
| Pavir.5NG491900.v4.1 | -3.1066 | 0.024601 | downstream target of AGL15-4 |
| Pavir.7KG242900.v4.1 | -3.1013 | 0.003822 | S-adenosyl-L-methionine-dependent methyltransferases superfamily protein |
| Pavir.5NG627200.v4.1 | -3.0859 | 0.001595 | Restriction endonuclease, type II-like superfamily protein |
| Pavir.5NG517700.v4.1 | -3.0827 | 0.002122 | Pyridoxal phosphate (PLP)-dependent transferases superfamily protein |
| Pavir.5KG686000.v4.1 | -3.079 | 7.11E-07 | Pectinacetylesterase family protein |
| Pavir.5NG236400.v4.1 | -3.0724 | 1.39E-06 | NUP50 (Nucleoporin 50 kDa) protein |
| Pavir.5NG478400.v4.1 | -3.0537 | 0.001463 | DNAJ heat shock N-terminal domain-containing protein |
| Pavir.7KG068200.v4.1 | -3.0535 | 0.00071 | ankyrin repeat family protein |
| Pavir.5KG724000.v4.1 | -3.0508 | 5.24E-05 |  |
| Pavir.3KG036800.v4.1 | -3.0076 | 9.12E-05 | chromatin assembly factor-1 (FASCIATA1) (FAS1) |
| Pavir.5NG516800.v4.1 | -2.9941 | 0.03965 | maternal effect embryo arrest 22 |
| Pavir.5NG271300.v4.1 | -2.9667 | 0.028037 | Cystathionine beta-synthase (CBS) protein |
| Pavir.5NG564300.v4.1 | -2.9593 | 1.83E-06 | Leucine-rich repeat protein kinase family protein |
| Pavir.6KG121500.v4.1 | -2.9583 | 0.010507 | S-adenosyl-L-methionine-dependent methyltransferases superfamily protein |
| Pavir.5NG459900.v4.1 | -2.9532 | 7.09E-06 | Microtubule associated protein (MAP65/ASE1) family protein |
| Pavir.8KG183800.v4.1 | -2.9468 | 0.00045 |  |
| Pavir.5NG520700.v4.1 | -2.9398 | 0.003061 | Polyketide cyclase / dehydrase and lipid transport protein |
| Pavir.5NG613400.v4.1 | -2.9346 | 0.000208 | basic helix-loop-helix (bHLH) DNA-binding superfamily protein |
| Pavir.5NG436300.v4.1 | -2.9239 | 0.000293 | Tetratricopeptide repeat (TPR)-like superfamily protein |
| Pavir.5NG535500.v4.1 | -2.9131 | 0.001662 | early nodulin-like protein 9 |
| Pavir.7KG297500.v4.1 | -2.9085 | 0.000168 | UDP-glucosyl transferase 85A2 |
| Pavir.5NG503600.v4.1 | -2.902 | 0.000392 |  |
| Pavir.7KG062400.v4.1 | -2.8942 | 0.017204 | inhibitor-3 |
| Pavir.9KG060700.v4.1 | -2.8606 | 0.027184 | Sodium Bile acid symporter family |
| Pavir.5NG359300.v4.1 | -2.8587 | 0.000985 | Leucine-rich repeat protein kinase family protein |
| Pavir.5NG487600.v4.1 | -2.8538 | 0.028316 |  |
| Pavir.5NG582100.v4.1 | -2.8443 | 0.003632 | ENTH/VHS family protein |
| Pavir.5NG553400.v4.1 | -2.8362 | 5.84E-06 | nodulin MtN21 /EamA-like transporter family protein |
| Pavir.5NG495100.v4.1 | -2.831 | 0.000134 | Dynamin related protein 5A |
| Pavir.7KG255700.v4.1 | -2.808 | 1.07E-05 | TCP family transcription factor |
| Pavir.5NG528800.v4.1 | -2.797 | 0.046474 | serine-rich protein-related |
| Pavir.6NG281700.v4.1 | -2.7902 | 0.001502 | carboxyesterase 13 |
| Pavir.J761600.v4.1 | -2.7896 | 0.000646 | beta-galactosidase 10 |
| Pavir.6NG316000.v4.1 | -2.7754 | 1.72E-05 | Ribosomal protein L32e |
| Pavir.5NG523000.v4.1 | -2.769 | 0.000178 | ARM repeat superfamily protein |
| Pavir.7KG099500.v4.1 | -2.7675 | 3.9E-05 | P-loop containing nucleoside triphosphate hydrolases superfamily protein |
| Pavir.5NG513400.v4.1 | -2.7608 | 0.015443 | calmodulin-like 41 |
| Pavir.6KG328800.v4.1 | -2.7557 | 0.001621 | GLN phosphoribosyl pyrophosphate amidotransferase 1 |
| Pavir.5NG582200.v4.1 | -2.7431 | 2.58E-05 | Fasciclin-like arabinogalactan family protein |
| Pavir.5NG011100.v4.1 | -2.7286 | 0.046879 | GATA transcription factor 17 |
| Pavir.6NG346700.v4.1 | -2.7247 | 0.000269 | NC domain-containing protein-related |
| Pavir.2KG317300.v4.1 | -2.7111 | 0.042898 | Calcium-dependent phosphotriesterase superfamily protein |
| Pavir.5NG616100.v4.1 | -2.6963 | 0.001465 | ARM repeat superfamily protein |
| Pavir.5NG641500.v4.1 | -2.6931 | 1.44E-05 | RNA binding Plectin/S10 domain-containing protein |
| Pavir.6KG328300.v4.1 | -2.6909 | 0.002041 | P-loop containing nucleoside triphosphate hydrolases superfamily protein |
| Pavir.7KG062600.v4.1 | -2.6683 | 0.031299 | DNA binding |
| Pavir.5NG459100.v4.1 | -2.6642 | 1.86E-05 | Transducin/WD40 repeat-like superfamily protein |
| Pavir.7KG158400.v4.1 | -2.6493 | 0.000182 |  |
| Pavir.5NG567600.v4.1 | -2.6363 | 2.41E-05 | Major facilitator superfamily protein |
| Pavir.2KG091800.v4.1 | -2.5878 | 0.002107 | HXXXD-type acyl-transferase family protein |
| Pavir.5NG565100.v4.1 | -2.5812 | 2.85E-05 | Bifunctional inhibitor/lipid-transfer protein/seed storage 2S albumin superfamily protein |
| Pavir.1NG250600.v4.1 | -2.5811 | 0.000288 | senescence-associated gene 29 |
| Pavir.5NG556800.v4.1 | -2.5805 | 0.024199 | Tetratricopeptide repeat (TPR)-like superfamily protein |
| Pavir.7KG178600.v4.1 | -2.5635 | 0.000843 | ChaC-like family protein |
| Pavir.5NG299900.v4.1 | -2.5621 | 0.013762 | ubiquitin-conjugating enzyme 37 |
| Pavir.7KG158800.v4.1 | -2.562 | 0.000734 | Disease resistance protein (CC-NBS-LRR class) family |
| Pavir.7KG133400.v4.1 | -2.5606 | 0.010907 | alpha/beta-Hydrolases superfamily protein |
| Pavir.5NG619500.v4.1 | -2.553 | 0.002259 | nuclear factor Y, subunit B5 |
| Pavir.2KG376700.v4.1 | -2.5526 | 0.0038 | glycosyl hydrolase 9B18 |
| Pavir.5NG554100.v4.1 | -2.548 | 0.001616 | ABA-responsive element binding protein 3 |
| Pavir.2NG354100.v4.1 | -2.5431 | 7.58E-05 | Adenine nucleotide alpha hydrolases-like superfamily protein |
| Pavir.5NG378900.v4.1 | -2.5364 | 0.002186 |  |
| Pavir.5NG521600.v4.1 | -2.5316 | 0.000885 |  |
| Pavir.5KG636500.v4.1 | -2.5261 | 0.014792 | Mitochondrial transcription termination factor family protein |
| Pavir.5KG633400.v4.1 | -2.5181 | 0.00055 | Bifunctional inhibitor/lipid-transfer protein/seed storage 2S albumin superfamily protein |
| Pavir.5NG108100.v4.1 | -2.5178 | 0.021507 | nucleobase-ascorbate transporter 12 |
| Pavir.5NG580200.v4.1 | -2.5131 | 0.000134 | FUS3-complementing gene 1 |
| Pavir.5NG272800.v4.1 | -2.5046 | 0.020645 | Carbohydrate-binding X8 domain superfamily protein |
| Pavir.5NG378200.v4.1 | -2.4998 | 8.4E-05 | Histone superfamily protein |
| Pavir.5NG638000.v4.1 | -2.4978 | 0.000134 | RNA-binding (RRM/RBD/RNP motifs) family protein |
| Pavir.6NG212900.v4.1 | -2.489 | 0.003584 | Protein of unknown function (DUF810) |
| Pavir.5NG452100.v4.1 | -2.4881 | 0.00033 | S-adenosyl-L-methionine-dependent methyltransferases superfamily protein |
| Pavir.5NG446700.v4.1 | -2.4846 | 0.000111 |  |
| Pavir.5NG644500.v4.1 | -2.4828 | 0.000251 | Small nuclear ribonucleoprotein family protein |
| Pavir.5NG499700.v4.1 | -2.4827 | 0.008532 | alpha/beta-Hydrolases superfamily protein |
| Pavir.5NG542600.v4.1 | -2.4802 | 0.000146 | Cyclin family protein |
| Pavir.7KG248100.v4.1 | -2.4786 | 0.044433 | Serinc-domain containing serine and sphingolipid biosynthesis protein |
| Pavir.J007100.v4.1 | -2.4762 | 0.028716 |  |
| Pavir.8KG360600.v4.1 | -2.472 | 0.002212 | Transmembrane amino acid transporter family protein |
| Pavir.5NG481800.v4.1 | -2.4708 | 0.002542 | UDP-glucosyl transferase 88A1 |
| Pavir.9KG480500.v4.1 | -2.4695 | 0.001384 | RNA-binding (RRM/RBD/RNP motifs) family protein |
| Pavir.8KG181200.v4.1 | -2.4675 | 0.000942 | Plant protein of unknown function (DUF639) |
| Pavir.5NG449000.v4.1 | -2.4665 | 0.000468 | AWPM-19-like family protein |
| Pavir.5NG058200.v4.1 | -2.4613 | 0.000533 | alpha/beta-Hydrolases superfamily protein |
| Pavir.1NG071900.v4.1 | -2.4601 | 0.005723 | squamosa promoter binding protein-like 8 |
| Pavir.5NG429900.v4.1 | -2.4565 | 0.025215 |  |
| Pavir.5KG633500.v4.1 | -2.4561 | 0.003238 | RHO guanyl-nucleotide exchange factor 7 |
| Pavir.4NG334500.v4.1 | -2.4531 | 0.006293 |  |
| Pavir.5NG489000.v4.1 | -2.4475 | 0.043764 | protein kinase family protein / peptidoglycan-binding LysM domain-containing protein |
| Pavir.J314500.v4.1 | -2.4464 | 0.005015 | zinc ion binding;DNA binding |
| Pavir.5NG436700.v4.1 | -2.4458 | 0.000398 | voltage dependent anion channel 1 |
| Pavir.5KG735800.v4.1 | -2.442 | 0.000139 |  |
| Pavir.7KG276800.v4.1 | -2.4411 | 0.035817 |  |
| Pavir.5NG611700.v4.1 | -2.4349 | 0.001988 | DHHC-type zinc finger family protein |
| Pavir.7KG212700.v4.1 | -2.4341 | 0.000881 | DNA-binding HORMA family protein |
| Pavir.5KG635900.v4.1 | -2.4307 | 0.000182 | alpha-glucan phosphorylase 2 |
| Pavir.3KG425200.v4.1 | -2.4256 | 0.016359 | Protein of unknown function (DUF594) |
| Pavir.5NG519700.v4.1 | -2.421 | 0.035512 | Transcription initiation factor IIF, beta subunit |
| Pavir.5NG448000.v4.1 | -2.4126 | 0.001767 | DNAJ heat shock N-terminal domain-containing protein |
| Pavir.5NG559900.v4.1 | -2.4124 | 0.000453 | Leucine-rich repeat protein kinase family protein |
| Pavir.5NG568300.v4.1 | -2.4112 | 0.006633 | PLAC8 family protein |
| Pavir.7KG223900.v4.1 | -2.4036 | 0.000281 |  |
| Pavir.5NG636900.v4.1 | -2.402 | 0.000897 | Protein of unknown function (DUF3411) |
| Pavir.3KG251300.v4.1 | -2.3999 | 0.000389 | Homeodomain-like superfamily protein |
| Pavir.5NG634300.v4.1 | -2.3997 | 0.002134 | FIZZY-related 3 |
| Pavir.5NG636300.v4.1 | -2.3922 | 0.000171 | Leucine-rich receptor-like protein kinase family protein |
| Pavir.6NG364700.v4.1 | -2.3884 | 0.001838 |  |
| Pavir.4KG396200.v4.1 | -2.3855 | 0.039406 | RNA-binding (RRM/RBD/RNP motifs) family protein |
| Pavir.5KG667500.v4.1 | -2.3725 | 0.004372 | K-box region and MADS-box transcription factor family protein |
| Pavir.5NG506800.v4.1 | -2.3678 | 0.000842 | Xanthine/uracil permease family protein |
| Pavir.5NG503400.v4.1 | -2.3653 | 0.000221 | Protein of unknown function, DUF642 |
| Pavir.1NG519000.v4.1 | -2.3593 | 0.018847 | CAP (Cysteine-rich secretory proteins, Antigen 5, and Pathogenesis-related 1 protein) superfamily protein |
| Pavir.2NG354400.v4.1 | -2.3586 | 0.020645 | beta-galactosidase 10 |
| Pavir.5NG587100.v4.1 | -2.3563 | 0.002976 | Nucleotidyltransferase family protein |
| Pavir.4NG165900.v4.1 | -2.351 | 0.00074 | cytochrome P450, family 94, subfamily D, polypeptide 1 |
| Pavir.J586900.v4.1 | -2.3492 | 0.001282 | RNA-binding (RRM/RBD/RNP motifs) family protein |
| Pavir.5NG279400.v4.1 | -2.3475 | 0.00498 | SLAC1 homologue 3 |
| Pavir.1NG266100.v4.1 | -2.3441 | 0.001209 | Protein of unknown function (DUF594) |
| Pavir.J057300.v4.1 | -2.3416 | 0.000615 | pfkB-like carbohydrate kinase family protein |
| Pavir.5NG480900.v4.1 | -2.3368 | 0.004531 | Ribosomal protein L20 |
| Pavir.6NG171100.v4.1 | -2.3364 | 0.000673 | UDP-glucosyl transferase 73B5 |
| Pavir.5NG110000.v4.1 | -2.3343 | 0.000235 | SNARE-like superfamily protein |
| Pavir.5NG420200.v4.1 | -2.3318 | 0.029146 |  |
| Pavir.5KG665000.v4.1 | -2.3246 | 0.000412 | Plant invertase/pectin methylesterase inhibitor superfamily |
| Pavir.9KG230000.v4.1 | -2.3235 | 0.00022 | ribosomal protein L5 B |
| Pavir.5NG386200.v4.1 | -2.3127 | 0.045075 |  |
| Pavir.5NG648000.v4.1 | -2.3072 | 0.016455 | Radical SAM superfamily protein |
| Pavir.5NG505300.v4.1 | -2.3068 | 0.000497 | phosphoenolpyruvate carboxylase 1 |
| Pavir.5NG495200.v4.1 | -2.3062 | 0.000516 | Homeodomain-like protein with RING/FYVE/PHD-type zinc finger domain |
| Pavir.2KG198800.v4.1 | -2.3055 | 0.007267 | amino acid permease 8 |
| Pavir.J104900.v4.1 | -2.3051 | 0.000885 | like COV 2 |
| Pavir.5NG543100.v4.1 | -2.302 | 0.000251 | beta-6 tubulin |
| Pavir.7KG241900.v4.1 | -2.3008 | 0.029311 | breast cancer associated RING 1 |
| Pavir.1KG250000.v4.1 | -2.2981 | 0.036551 | terpene synthase 21 |
| Pavir.5NG645700.v4.1 | -2.2904 | 0.000393 | UDP-D-apiose/UDP-D-xylose synthase 2 |
| Pavir.5NG495800.v4.1 | -2.2893 | 0.001362 | Integrin-linked protein kinase family |
| Pavir.5NG472100.v4.1 | -2.284 | 0.001582 | Hyaluronan / mRNA binding family |
| Pavir.5NG506500.v4.1 | -2.2727 | 0.001282 | CBL-interacting protein kinase 18 |
| Pavir.3KG478800.v4.1 | -2.2695 | 0.021291 | Nucleotide-diphospho-sugar transferases superfamily protein |
| Pavir.5NG058700.v4.1 | -2.2677 | 0.005807 | TBP-associated factor 7 |
| Pavir.7NG124200.v4.1 | -2.2607 | 0.001675 | Oxidoreductase, zinc-binding dehydrogenase family protein |
| Pavir.5NG491200.v4.1 | -2.2567 | 0.01475 | Nucleotidylyl transferase superfamily protein |
| Pavir.5KG737200.v4.1 | -2.2498 | 0.000897 | Calmodulin-binding transcription activator protein with CG-1 and Ankyrin domains |
| Pavir.5NG500600.v4.1 | -2.2415 | 0.002034 | GroES-like zinc-binding alcohol dehydrogenase family protein |
| Pavir.6NG300200.v4.1 | -2.2403 | 0.025191 | Mitochondrial substrate carrier family protein |
| Pavir.5KG634400.v4.1 | -2.2351 | 0.00498 | Arabidopsis Inositol phosphorylceramide synthase 1 |
| Pavir.5KG726600.v4.1 | -2.235 | 0.00153 |  |
| Pavir.5NG409800.v4.1 | -2.2334 | 0.005256 | kinesin like protein for actin based chloroplast movement 1 |
| Pavir.7KG273300.v4.1 | -2.2258 | 0.004789 | Domain of unknown function (DUF3598) |
| Pavir.7KG007500.v4.1 | -2.2184 | 0.013043 | kinase interacting family protein |
| Pavir.5NG510600.v4.1 | -2.2177 | 0.005849 | Glutathione S-transferase family protein |
| Pavir.5NG581900.v4.1 | -2.2165 | 0.003189 | Ribosomal protein L36e family protein |
| Pavir.6NG323500.v4.1 | -2.2152 | 0.00197 | P-loop containing nucleoside triphosphate hydrolases superfamily protein |
| Pavir.5NG528200.v4.1 | -2.2143 | 0.001349 | AAA-type ATPase family protein |
| Pavir.5NG300200.v4.1 | -2.2134 | 0.012355 | ATP binding microtubule motor family protein |
| Pavir.9NG688300.v4.1 | -2.2111 | 0.004554 |  |
| Pavir.4KG359000.v4.1 | -2.2099 | 0.00158 |  |
| Pavir.5NG522800.v4.1 | -2.1963 | 0.008246 | Protein kinase family protein |
| Pavir.J354200.v4.1 | -2.1958 | 0.000726 | SEC14 cytosolic factor family protein / phosphoglyceride transfer family protein |
| Pavir.5NG426200.v4.1 | -2.1952 | 0.015009 | Pentatricopeptide repeat (PPR) superfamily protein |
| Pavir.5NG511100.v4.1 | -2.1939 | 0.009537 | chorismate mutase 3 |
| Pavir.7KG068000.v4.1 | -2.1926 | 0.014675 | Ankyrin repeat family protein |
| Pavir.5NG582700.v4.1 | -2.1833 | 0.001237 | triosephosphate isomerase |
| Pavir.5KG664200.v4.1 | -2.1767 | 0.016356 | SET domain-containing protein |
| Pavir.5NG548300.v4.1 | -2.1759 | 0.002789 |  |
| Pavir.5NG428400.v4.1 | -2.1665 | 0.022384 | RING/U-box superfamily protein |
| Pavir.J477700.v4.1 | -2.1632 | 0.019674 | structural constituent of nuclear pore |
| Pavir.5NG473000.v4.1 | -2.158 | 0.00498 | hexokinase 1 |
| Pavir.6NG336900.v4.1 | -2.1529 | 0.001339 | general regulatory factor 7 |
| Pavir.5NG556500.v4.1 | -2.1523 | 0.001384 | ribosomal protein L24 |
| Pavir.5NG536400.v4.1 | -2.1522 | 0.007337 | pentatricopeptide repeat 336 |
| Pavir.3NG016000.v4.1 | -2.1506 | 0.047659 | xyloglucan xylosyltransferase 5 |
| Pavir.5NG507600.v4.1 | -2.1495 | 0.02628 | Tesmin/TSO1-like CXC domain-containing protein |
| Pavir.5NG499800.v4.1 | -2.1494 | 0.020075 |  |
| Pavir.J313400.v4.1 | -2.1485 | 0.001628 | myb domain protein 86 |
| Pavir.5NG410600.v4.1 | -2.1464 | 0.001903 | ubiquitin conjugating enzyme 9 |
| Pavir.7KG208600.v4.1 | -2.1458 | 0.014853 | AAA-type ATPase family protein |
| Novel00721 | -2.1443 | 0.004656 | - |
| Pavir.5NG504000.v4.1 | -2.1403 | 0.001464 | P-loop containing nucleoside triphosphate hydrolases superfamily protein |
| Pavir.5NG461500.v4.1 | -2.1373 | 0.004682 | P-loop containing nucleoside triphosphate hydrolases superfamily protein |
| Pavir.5NG458400.v4.1 | -2.1364 | 0.01992 | alpha/beta-Hydrolases superfamily protein |
| Pavir.5NG435700.v4.1 | -2.1352 | 0.001384 | IQ-domain 28 |
| Pavir.5KG065800.v4.1 | -2.134 | 0.004033 | gibberellin 3-oxidase 1 |
| Pavir.5KG615300.v4.1 | -2.132 | 0.006391 | NAD(P)-linked oxidoreductase superfamily protein |
| Pavir.6KG330500.v4.1 | -2.129 | 0.009368 | C2H2 and C2HC zinc fingers superfamily protein |
| Pavir.5NG500500.v4.1 | -2.1287 | 0.036668 |  |
| Pavir.J522300.v4.1 | -2.1285 | 0.005723 | Ribosomal protein L36e family protein |
| Pavir.5NG546200.v4.1 | -2.1285 | 0.001538 | Transmembrane proteins 14C |
| Pavir.7KG174500.v4.1 | -2.1256 | 0.016821 | alfin-like 3 |
| Pavir.J453100.v4.1 | -2.119 | 0.016455 | AAA-type ATPase family protein |
| Pavir.7KG112700.v4.1 | -2.1141 | 0.013606 |  |
| Pavir.J486100.v4.1 | -2.1106 | 0.017664 | translocase outer membrane 20-2 |
| Pavir.5NG443500.v4.1 | -2.109 | 0.002185 | Ribosomal L28e protein family |
| Pavir.5NG439500.v4.1 | -2.1073 | 0.001516 | Transducin family protein / WD-40 repeat family protein |
| Pavir.5NG493200.v4.1 | -2.1031 | 0.006058 | staurosporin and temperature sensitive 3-like A |
| Pavir.5NG541300.v4.1 | -2.101 | 0.002082 | DNA glycosylase superfamily protein |
| Pavir.7KG191500.v4.1 | -2.0975 | 0.003978 | receptor-like kinase 1 |
| Pavir.7KG257700.v4.1 | -2.0955 | 0.02955 |  |
| Pavir.5NG505800.v4.1 | -2.0935 | 0.003207 | Protein of unknown function (DUF688) |
| Pavir.5NG587300.v4.1 | -2.0917 | 0.02708 | S-adenosyl-L-methionine-dependent methyltransferases superfamily protein |
| Pavir.5NG272500.v4.1 | -2.0912 | 0.012377 | aspartic proteinase A1 |
| Pavir.5NG411000.v4.1 | -2.0893 | 0.002601 | Galactose mutarotase-like superfamily protein |
| Pavir.6NG315100.v4.1 | -2.0884 | 0.008754 | squamosa promoter binding protein-like 9 |
| Pavir.J546000.v4.1 | -2.0832 | 0.019737 | C2H2 and C2HC zinc fingers superfamily protein |
| Pavir.5KG727600.v4.1 | -2.0831 | 0.004288 | x-ray induced transcript 1 |
| Pavir.5NG644200.v4.1 | -2.0797 | 0.026274 | Leucine-rich repeat (LRR) family protein |
| Pavir.5NG426900.v4.1 | -2.0774 | 0.018847 | Nucleotidyltransferase family protein |
| Pavir.5KG634500.v4.1 | -2.0755 | 0.004287 | RING/FYVE/PHD zinc finger superfamily protein |
| Pavir.5NG619900.v4.1 | -2.0744 | 0.003488 | K+ uptake transporter 3 |
| Pavir.5NG576700.v4.1 | -2.0698 | 0.002158 | Ribosomal protein S12/S23 family protein |
| Pavir.5NG631000.v4.1 | -2.0621 | 0.006371 | uridine 5\'-monophosphate synthase / UMP synthase (PYRE-F) (UMPS) |
| Pavir.5NG379800.v4.1 | -2.061 | 0.008854 | voltage dependent anion channel 1 |
| Pavir.5NG569400.v4.1 | -2.0602 | 0.00388 | prenylated RAB acceptor 1.B4 |
| Pavir.5NG574400.v4.1 | -2.0585 | 0.003156 | calcium-dependent protein kinase 13 |
| Pavir.5NG505400.v4.1 | -2.055 | 0.004384 | cytidinediphosphate diacylglycerol synthase 2 |
| Pavir.5NG622800.v4.1 | -2.0544 | 0.003872 | RNA-binding protein-defense related 1 |
| Pavir.5KG723400.v4.1 | -2.0537 | 0.048614 | Protein of unknown function, DUF538 |
| Pavir.7KG216100.v4.1 | -2.0533 | 0.01885 |  |
| Pavir.5NG110300.v4.1 | -2.0528 | 0.011421 | BolA-like family protein |
| Pavir.7KG197900.v4.1 | -2.0505 | 0.006441 | kinesin-like protein 1 |
| Pavir.5NG641700.v4.1 | -2.0464 | 0.029557 | sulfotransferase 17 |
| Pavir.5KG656500.v4.1 | -2.038 | 0.035487 | UDP-Glycosyltransferase superfamily protein |
| Pavir.7KG205500.v4.1 | -2.0369 | 0.007786 | eukaryotic translation initiation factor 4B1 |
| Pavir.5NG480300.v4.1 | -2.0357 | 0.003501 | translocase of outer membrane 22-V |
| Pavir.5NG529300.v4.1 | -2.0357 | 0.016601 |  |
| Pavir.2KG387500.v4.1 | -2.0317 | 0.02245 | plasma membrane intrinsic protein 2;5 |
| Pavir.7KG268800.v4.1 | -2.0276 | 0.003083 | aldehyde dehydrogenase 3F1 |
| Pavir.5NG548200.v4.1 | -2.0269 | 0.026404 | myb domain protein 33 |
| Pavir.2KG578500.v4.1 | -2.0246 | 0.002681 | Eukaryotic aspartyl protease family protein |
| Pavir.5NG590300.v4.1 | -2.021 | 0.009653 |  |
| Pavir.J496400.v4.1 | -2.0202 | 0.003789 | formin homology 1 |
| Pavir.5NG575400.v4.1 | -2.0186 | 0.034516 | serine carboxypeptidase-like 27 |
| Pavir.5NG451300.v4.1 | -2.0178 | 0.003896 | ATP binding cassette subfamily B4 |
| Pavir.3KG477800.v4.1 | -2.0167 | 0.017464 | response regulator 11 |
| Pavir.5NG260300.v4.1 | -2.0082 | 0.049438 |  |
| Pavir.5NG439700.v4.1 | -2.0081 | 0.004033 | Protein kinase superfamily protein |
| Pavir.5NG414000.v4.1 | -2.0079 | 0.005887 | Eukaryotic aspartyl protease family protein |
| Pavir.5NG644900.v4.1 | -2.0067 | 0.009849 | proteinaceous RNase P 2 |
| Pavir.5NG501200.v4.1 | -1.9996 | 0.004288 | Transcriptional factor B3 family protein / auxin-responsive factor AUX/IAA-related |
| Pavir.5NG459800.v4.1 | -1.992 | 0.00498 |  |
| Pavir.5NG272700.v4.1 | -1.9918 | 0.010228 | Protein kinase superfamily protein |
| Pavir.9NG563000.v4.1 | -1.989 | 0.017372 |  |
| Pavir.5NG429700.v4.1 | -1.9882 | 0.007267 |  |
| Pavir.9NG674900.v4.1 | -1.9861 | 0.041855 |  |
| Pavir.5NG506700.v4.1 | -1.9851 | 0.012667 | transcriptional regulator family protein |
| Pavir.5NG426500.v4.1 | -1.9844 | 0.012667 | glucan synthase-like 5 |
| Pavir.7KG220400.v4.1 | -1.9821 | 0.003592 | B-box zinc finger family protein |
| Pavir.5NG107300.v4.1 | -1.9813 | 0.008892 | Carbohydrate-binding-like fold |
| Pavir.1KG422500.v4.1 | -1.979 | 0.011173 | glutathione S-transferase TAU 18 |
| Pavir.5NG577600.v4.1 | -1.9786 | 0.006369 | Ypt/Rab-GAP domain of gyp1p superfamily protein |
| Pavir.5NG481200.v4.1 | -1.9693 | 0.007674 | respiratory burst oxidase protein F |
| Pavir.5NG406700.v4.1 | -1.9691 | 0.003789 | ketol-acid reductoisomerase |
| Pavir.5NG262900.v4.1 | -1.9684 | 0.012557 | Metal-dependent phosphohydrolase |
| Pavir.6NG311500.v4.1 | -1.9674 | 0.005798 | cyclin-dependent kinase B2;1 |
| Pavir.5NG619700.v4.1 | -1.965 | 0.047216 | cullin 1 |
| Pavir.5NG589300.v4.1 | -1.9617 | 0.018992 | sulfate transporter 3;1 |
| Pavir.5NG406900.v4.1 | -1.9617 | 0.004489 | TRICHOME BIREFRINGENCE-LIKE 19 |
| Pavir.5NG566800.v4.1 | -1.9592 | 0.019737 | allergen-related |
| Pavir.1KG147700.v4.1 | -1.9511 | 0.025518 | xyloglucan endotransglucosylase/hydrolase 12 |
| Pavir.5NG483400.v4.1 | -1.9399 | 0.004771 | VIRB2-interacting protein 1 |
| Pavir.1NG467400.v4.1 | -1.9382 | 0.048583 | FASCICLIN-like arabinogalactan 2 |
| Pavir.5NG491800.v4.1 | -1.9368 | 0.006391 | P-loop nucleoside triphosphate hydrolases superfamily protein with CH (Calponin Homology) domain |
| Pavir.5NG613200.v4.1 | -1.9364 | 0.004598 | auxin response factor 2 |
| Pavir.5NG438600.v4.1 | -1.9363 | 0.023588 | calcineurin B-like protein 10 |
| Pavir.1KG126000.v4.1 | -1.9291 | 0.030114 |  |
| Pavir.5NG430100.v4.1 | -1.9264 | 0.011845 | indoleacetic acid-induced protein 8 |
| Pavir.5NG622500.v4.1 | -1.9257 | 0.006371 | arabinogalactan protein 26 |
| Pavir.1KG476500.v4.1 | -1.9215 | 0.006894 | Phosphofructokinase family protein |
| Pavir.5NG552900.v4.1 | -1.9211 | 0.004905 | Drug/metabolite transporter superfamily protein |
| Pavir.5NG265500.v4.1 | -1.9198 | 0.006391 | plastidic pyruvate kinase beta subunit 1 |
| Pavir.J354700.v4.1 | -1.9147 | 0.007877 | serine hydroxymethyltransferase 7 |
| Pavir.5NG532100.v4.1 | -1.9134 | 0.00908 | Adenine nucleotide alpha hydrolases-like superfamily protein |
| Pavir.5NG479000.v4.1 | -1.913 | 0.022384 | AAA-type ATPase family protein |
| Pavir.5NG441500.v4.1 | -1.9077 | 0.012055 | cytidine deaminase 1 |
| Pavir.5NG589800.v4.1 | -1.9054 | 0.038191 | Alcohol dehydrogenase transcription factor Myb/SANT-like family protein |
| Pavir.9NG564000.v4.1 | -1.9003 | 0.032905 | Phosphotyrosine protein phosphatases superfamily protein |
| Pavir.7KG202000.v4.1 | -1.9 | 0.04309 | UV-B-insensitive 4-like |
| Pavir.6NG270400.v4.1 | -1.8982 | 0.020544 | D6 protein kinase like 2 |
| Pavir.2KG302800.v4.1 | -1.8926 | 0.007786 | xyloglucan endotransglucosylase/hydrolase 28 |
| Pavir.5NG519200.v4.1 | -1.8918 | 0.015551 |  |
| Pavir.5NG473300.v4.1 | -1.8899 | 0.007013 | Ribosomal protein S24e family protein |
| Pavir.8KG182900.v4.1 | -1.8846 | 0.016455 | sorting nexin 1 |
| Pavir.5NG543200.v4.1 | -1.8836 | 0.032591 | Ubiquitin-associated (UBA) protein |
| Pavir.1KG491500.v4.1 | -1.8779 | 0.017763 | Protein kinase superfamily protein |
| Pavir.7NG142200.v4.1 | -1.8778 | 0.02376 |  |
| Pavir.5KG635000.v4.1 | -1.8768 | 0.011119 | SOUL heme-binding family protein |
| Pavir.6NG280000.v4.1 | -1.8752 | 0.01475 | homeobox protein 16 |
| Pavir.5NG645600.v4.1 | -1.8749 | 0.017372 |  |
| Pavir.5NG552100.v4.1 | -1.8727 | 0.037184 |  |
| Pavir.5NG404900.v4.1 | -1.8713 | 0.009368 | SWAP (Suppressor-of-White-APricot)/surp domain-containing protein / ubiquitin family protein |
| Pavir.5NG553300.v4.1 | -1.871 | 0.007376 | cytochrome P450, family 98, subfamily A, polypeptide 3 |
| Pavir.7KG192500.v4.1 | -1.8688 | 0.028316 | anthranilate synthase beta subunit 1 |
| Pavir.4NG281100.v4.1 | -1.865 | 0.009025 | Pathogenesis-related thaumatin superfamily protein |
| Pavir.5NG501800.v4.1 | -1.8601 | 0.016267 | TCP family transcription factor 4 |
| Pavir.7KG027300.v4.1 | -1.8541 | 0.010771 | multidrug resistance-associated protein 4 |
| Pavir.3KG477700.v4.1 | -1.8493 | 0.045095 |  |
| Pavir.7KG116100.v4.1 | -1.8479 | 0.025978 | Small nuclear ribonucleoprotein family protein |
| Pavir.5NG492000.v4.1 | -1.8473 | 0.009225 | villin 2 |
| Pavir.5NG308300.v4.1 | -1.8472 | 0.016455 | tetratricopetide-repeat thioredoxin-like 1 |
| Pavir.5NG646400.v4.1 | -1.846 | 0.029311 | Drought-responsive family protein |
| Pavir.5NG442800.v4.1 | -1.8454 | 0.012849 | myristoyl-CoA:protein N-myristoyltransferase |
| Pavir.5NG634100.v4.1 | -1.8452 | 0.013662 | Nucleic acid-binding, OB-fold-like protein |
| Pavir.7KG177700.v4.1 | -1.842 | 0.012305 | glutamate decarboxylase 2 |
| Pavir.5NG436800.v4.1 | -1.8394 | 0.036739 | alpha-amylase-like 3 |
| Pavir.5NG457900.v4.1 | -1.8387 | 0.016572 |  |
| Pavir.5NG494600.v4.1 | -1.8361 | 0.045075 | pyrimidin 4 |
| Pavir.5NG645900.v4.1 | -1.834 | 0.017372 | cellulose synthase like G3 |
| Pavir.5NG479300.v4.1 | -1.8336 | 0.01164 |  |
| Pavir.5NG011000.v4.1 | -1.8304 | 0.028438 | P-glycoprotein 20 |
| Pavir.5NG352200.v4.1 | -1.8281 | 0.041579 | ATP binding microtubule motor family protein |
| Pavir.8KG099900.v4.1 | -1.8252 | 0.023322 | GAST1 protein homolog 3 |
| Pavir.5NG620500.v4.1 | -1.8245 | 0.020275 | Protein kinase superfamily protein |
| Pavir.5NG139900.v4.1 | -1.8232 | 0.040988 | ubiquitin-conjugating enzyme 27 |
| Pavir.3KG015100.v4.1 | -1.8186 | 0.042357 | carboxyesterase 16 |
| Pavir.5NG311100.v4.1 | -1.8186 | 0.019935 | sterol 4-alpha-methyl-oxidase 2-2 |
| Pavir.5NG610700.v4.1 | -1.8166 | 0.029146 | ENTH/VHS family protein |
| Pavir.1NG233900.v4.1 | -1.8138 | 0.013035 | S-adenosyl-L-methionine-dependent methyltransferases superfamily protein |
| Pavir.5NG460000.v4.1 | -1.8123 | 0.010931 | ATP synthase alpha/beta family protein |
| Pavir.5NG410700.v4.1 | -1.8091 | 0.023371 | E2F/DP family winged-helix DNA-binding domain |
| Pavir.6NG323300.v4.1 | -1.8053 | 0.023682 | Thioredoxin family protein |
| Pavir.5NG549200.v4.1 | -1.8032 | 0.012696 | Protein of unknown function (DUF3411) |
| Pavir.5NG567900.v4.1 | -1.8022 | 0.01583 | succinate dehydrogenase 5 |
| Pavir.5NG275100.v4.1 | -1.7961 | 0.011869 | MAP kinase 20 |
| Pavir.J719500.v4.1 | -1.7954 | 0.013319 | ROP interactive partner 5 |
| Pavir.6NG329000.v4.1 | -1.7927 | 0.039465 |  |
| Pavir.5NG551700.v4.1 | -1.7923 | 0.02891 | apurinic endonuclease-redox protein |
| Pavir.2NG354200.v4.1 | -1.7916 | 0.029146 | ubiquitin family protein |
| Pavir.5NG528600.v4.1 | -1.7903 | 0.016267 | SNARE associated Golgi protein family |
| Pavir.7KG156600.v4.1 | -1.7869 | 0.014815 | RNA binding Plectin/S10 domain-containing protein |
| Pavir.5NG635400.v4.1 | -1.7864 | 0.016433 | non-specific phospholipase C2 |
| Pavir.1KG200600.v4.1 | -1.7861 | 0.01807 | arabinogalactan protein 22 |
| Pavir.7KG282300.v4.1 | -1.782 | 0.049376 | Nucleotide-diphospho-sugar transferases superfamily protein |
| Pavir.5NG262500.v4.1 | -1.78 | 0.041855 | prenylcysteine methylesterase |
| Pavir.5NG434600.v4.1 | -1.7798 | 0.039465 |  |
| Pavir.5KG666300.v4.1 | -1.7786 | 0.015661 | Nodulin MtN3 family protein |
| Pavir.5NG436200.v4.1 | -1.7771 | 0.024294 |  |
| Pavir.5KG632800.v4.1 | -1.772 | 0.031022 | prenylated RAB acceptor 1.A2 |
| Pavir.8KG180800.v4.1 | -1.7694 | 0.039682 | DNA-directed DNA polymerases |
| Pavir.5NG558600.v4.1 | -1.7684 | 0.019737 | Auxin efflux carrier family protein |
| Pavir.7KG147800.v4.1 | -1.7678 | 0.024729 | Histone superfamily protein |
| Pavir.5KG007600.v4.1 | -1.7654 | 0.016328 | VIRB2-interacting protein 1 |
| Pavir.5NG579800.v4.1 | -1.7621 | 0.028125 | P-loop containing nucleoside triphosphate hydrolases superfamily protein |
| Pavir.5KG687000.v4.1 | -1.7597 | 0.032145 | pfkB-like carbohydrate kinase family protein |
| Pavir.6NG368500.v4.1 | -1.7586 | 0.034806 | 31-kDa RNA binding protein |
| Pavir.5NG546300.v4.1 | -1.7576 | 0.022713 | Nucleic acid-binding, OB-fold-like protein |
| Pavir.5NG427000.v4.1 | -1.7555 | 0.041054 | Protein of unknown function (DUF1421) |
| Pavir.5NG467200.v4.1 | -1.7533 | 0.016351 | flavodoxin-like quinone reductase 1 |
| Pavir.5NG624300.v4.1 | -1.7518 | 0.028316 | hexokinase-like 1 |
| Pavir.5NG443700.v4.1 | -1.7501 | 0.032989 | transcriptional coactivator p15 (PC4) family protein (KELP) |
| Pavir.5NG572200.v4.1 | -1.7471 | 0.024427 | Plant protein of unknown function (DUF828) |
| Pavir.2NG645700.v4.1 | -1.7465 | 0.020194 | RNA-binding (RRM/RBD/RNP motifs) family protein |
| Pavir.5NG440800.v4.1 | -1.7458 | 0.046565 | RNA-binding KH domain-containing protein |
| Pavir.5NG638600.v4.1 | -1.7427 | 0.020527 | arginine/serine-rich 45 |
| Pavir.7KG224900.v4.1 | -1.7399 | 0.01992 | Ferritin/ribonucleotide reductase-like family protein |
| Pavir.5NG627700.v4.1 | -1.7378 | 0.028316 | pyrroline-5- carboxylate (P5C) reductase |
| Pavir.5NG265000.v4.1 | -1.7371 | 0.022713 | DEK domain-containing chromatin associated protein |
| Pavir.1NG398500.v4.1 | -1.7294 | 0.035808 | GDSL-like Lipase/Acylhydrolase superfamily protein |
| Pavir.5NG409000.v4.1 | -1.727 | 0.030088 | U2 snRNP auxilliary factor, large subunit, splicing factor |
| Pavir.5NG447500.v4.1 | -1.7269 | 0.031194 | farnesyl diphosphate synthase 1 |
| Pavir.5NG010400.v4.1 | -1.7263 | 0.02955 |  |
| Pavir.5NG420100.v4.1 | -1.7253 | 0.044433 | Magnesium transporter CorA-like family protein |
| Pavir.7KG193600.v4.1 | -1.7242 | 0.02891 |  |
| Pavir.5NG012400.v4.1 | -1.7231 | 0.026496 | sister chromatid cohesion 1 protein 4 |
| Pavir.5NG571700.v4.1 | -1.7217 | 0.030871 | RING/U-box superfamily protein |
| Pavir.5NG576200.v4.1 | -1.7216 | 0.028592 | ROP interactive partner 3 |
| Pavir.5NG644300.v4.1 | -1.7174 | 0.031335 | Vacuolar protein sorting 55 (VPS55) family protein |
| Pavir.5NG622700.v4.1 | -1.7166 | 0.046565 | photosystem II reaction center PSB28 protein |
| Pavir.5KG725900.v4.1 | -1.7118 | 0.041152 | Pre-rRNA-processing protein TSR2, conserved region |
| Pavir.7KG118600.v4.1 | -1.711 | 0.042221 | Protein kinase superfamily protein |
| Pavir.6NG324100.v4.1 | -1.7099 | 0.02528 | Ribosomal protein S25 family protein |
| Pavir.5NG617400.v4.1 | -1.7083 | 0.031238 | Transducin/WD40 repeat-like superfamily protein |
| Pavir.5NG585500.v4.1 | -1.7072 | 0.032781 | DHHC-type zinc finger family protein |
| Pavir.3NG223500.v4.1 | -1.7065 | 0.031022 | polyol/monosaccharide transporter 5 |
| Pavir.5NG425700.v4.1 | -1.7065 | 0.031299 | Transcriptional factor B3 family protein / auxin-responsive factor AUX/IAA-related |
| Pavir.5NG580700.v4.1 | -1.7056 | 0.024374 |  |
| Pavir.1NG186100.v4.1 | -1.7048 | 0.025691 | arabinogalactan protein 16 |
| Pavir.5NG421000.v4.1 | -1.7001 | 0.025388 | Eukaryotic translation initiation factor 3 subunit 7 (eIF-3) |
| Pavir.5KG726400.v4.1 | -1.6888 | 0.043118 |  |
| Pavir.5KG731300.v4.1 | -1.6871 | 0.032324 | plant U-box 45 |
| Pavir.7KG242400.v4.1 | -1.6865 | 0.048134 | RING/U-box superfamily protein |
| Pavir.6NG261100.v4.1 | -1.6861 | 0.033268 | FASCICLIN-like arabinoogalactan 7 |
| Pavir.5NG501600.v4.1 | -1.6858 | 0.033488 | glucan synthase-like 1 |
| Pavir.9KG654600.v4.1 | -1.6788 | 0.028809 | Ribosomal protein L6 family protein |
| Pavir.5NG270100.v4.1 | -1.6753 | 0.044258 | Pectin lyase-like superfamily protein |
| Pavir.5NG613500.v4.1 | -1.6712 | 0.029724 |  |
| Pavir.5NG558400.v4.1 | -1.6684 | 0.028037 | Phosphoglycerate mutase, 2,3-bisphosphoglycerate-independent |
| Pavir.5NG492800.v4.1 | -1.6676 | 0.038564 | DNA binding;zinc ion binding;nucleic acid binding;nucleic acid binding |
| Pavir.5NG575900.v4.1 | -1.6633 | 0.033576 | IQ-domain 2 |
| Pavir.5NG620600.v4.1 | -1.6618 | 0.027926 | 6-phosphogluconate dehydrogenase family protein |
| Pavir.5NG490100.v4.1 | -1.6579 | 0.02665 | Ribosomal protein S5/Elongation factor G/III/V family protein |
| Pavir.3KG284400.v4.1 | -1.6567 | 0.039406 |  |
| Pavir.5NG455400.v4.1 | -1.6564 | 0.037211 | flower-specific, phytochrome-associated protein phosphatase 3 |
| Pavir.5NG642500.v4.1 | -1.6505 | 0.027448 | actin 7 |
| Pavir.5NG264400.v4.1 | -1.6494 | 0.045772 | HCP-like superfamily protein with MYND-type zinc finger |
| Pavir.5KG576300.v4.1 | -1.6447 | 0.045909 | receptor kinase 3 |
| Pavir.5NG624000.v4.1 | -1.6441 | 0.033092 | S-formylglutathione hydrolase |
| Pavir.5NG574700.v4.1 | -1.6436 | 0.046565 | Protein kinase superfamily protein |
| Pavir.5NG452400.v4.1 | -1.6422 | 0.04333 | ENTH/ANTH/VHS superfamily protein |
| Pavir.5NG364400.v4.1 | -1.6391 | 0.045244 |  |
| Pavir.8KG184900.v4.1 | -1.6374 | 0.036493 | NIMA (never in mitosis, gene A)-related 6 |
| Pavir.7KG186800.v4.1 | -1.6342 | 0.035547 | ATP binding cassette subfamily B19 |
| Pavir.1NG408000.v4.1 | -1.634 | 0.049837 | glycosyl hydrolase 9B1 |
| Pavir.9NG270500.v4.1 | -1.6317 | 0.041221 |  |
| Pavir.5NG501500.v4.1 | -1.6304 | 0.043425 | Nucleic acid-binding, OB-fold-like protein |
| Pavir.5NG648200.v4.1 | -1.6302 | 0.045095 | homolog of mamallian P58IPK |
| Pavir.5NG560000.v4.1 | -1.6302 | 0.038209 | ubiquitin-conjugating enzyme 11 |
| Pavir.5NG552000.v4.1 | -1.6296 | 0.03933 | 6-phosphogluconate dehydrogenase family protein |
| Pavir.9KG643000.v4.1 | -1.6267 | 0.033806 | myo-inositol-1-phosphate synthase 2 |
| Pavir.5NG645800.v4.1 | -1.6266 | 0.046786 | eukaryotic translation initiation factor 4E |
| Pavir.5NG503200.v4.1 | -1.6246 | 0.043923 | RNA-binding protein |
| Pavir.5NG494000.v4.1 | -1.6229 | 0.036716 | Glycosyl hydrolase superfamily protein |
| Pavir.7KG282200.v4.1 | -1.6213 | 0.049438 | Pectin lyase-like superfamily protein |
| Pavir.5NG590200.v4.1 | -1.6173 | 0.037255 | Leucine-rich receptor-like protein kinase family protein |
| Pavir.7NG382700.v4.1 | -1.6165 | 0.035808 | N-terminal nucleophile aminohydrolases (Ntn hydrolases) superfamily protein |
| Pavir.5NG588800.v4.1 | -1.615 | 0.035899 | NADH-ubiquinone oxidoreductase 20 kDa subunit, mitochondrial |
| Pavir.4KG370300.v4.1 | -1.6105 | 0.04587 | Sec14p-like phosphatidylinositol transfer family protein |
| Pavir.9NG767700.v4.1 | -1.6063 | 0.039969 | glutathione S-transferase phi 8 |
| Pavir.5NG626600.v4.1 | -1.6039 | 0.045909 | RNA-binding (RRM/RBD/RNP motifs) family protein |
| Pavir.5NG058600.v4.1 | -1.5991 | 0.039211 | RAN GTPase 3 |
| Pavir.7KG229100.v4.1 | -1.5906 | 0.046729 | ribosomal protein L23AB |
| Pavir.5NG526800.v4.1 | -1.5845 | 0.049967 | Vps51/Vps67 family (components of vesicular transport) protein |
| Pavir.5NG010900.v4.1 | -1.5834 | 0.047406 | SAND family protein |
| Pavir.2KG198900.v4.1 | -1.5808 | 0.044433 | catalytic LigB subunit of aromatic ring-opening dioxygenase family |
| Pavir.3KG478700.v4.1 | -1.567 | 0.04309 | Aldolase superfamily protein |
| Pavir.5NG415700.v4.1 | -1.5605 | 0.047216 | NADH-dependent glutamate synthase 1 |
| Pavir.J239000.v4.1 | -1.556 | 0.049837 |  |
| Pavir.4KG379600.v4.1 | 1.6369 | 0.035531 | P-loop containing nucleoside triphosphate hydrolases superfamily protein |
| Pavir.2KG329300.v4.1 | 1.6524 | 0.049106 |  |
| Pavir.1NG010500.v4.1 | 1.6587 | 0.02748 | catalase 1 |
| Pavir.J175400.v4.1 | 1.6588 | 0.032781 |  |
| Pavir.8NG160400.v4.1 | 1.6702 | 0.034516 | cysteine-rich RLK (RECEPTOR-like protein kinase) 40 |
| Pavir.2NG429900.v4.1 | 1.6843 | 0.033276 |  |
| Pavir.9NG745800.v4.1 | 1.6856 | 0.028316 | phosphate transporter 1;7 |
| Pavir.3KG042200.v4.1 | 1.7294 | 0.028316 | lipid transfer protein 12 |
| Pavir.9NG509200.v4.1 | 1.7478 | 0.020555 | Peroxidase superfamily protein |
| Pavir.6NG224300.v4.1 | 1.757 | 0.038199 | Phosphofructokinase family protein |
| Pavir.9NG786400.v4.1 | 1.7724 | 0.015287 | Hypoxia-responsive family protein |
| Pavir.8KG022000.v4.1 | 1.7797 | 0.010049 | lipid transfer protein 12 |
| Pavir.6KG008200.v4.1 | 1.7906 | 0.043118 | cytochrome P450, family 71, subfamily A, polypeptide 22 |
| Pavir.4KG265100.v4.1 | 1.807 | 0.009996 | protodermal factor 1 |
| Pavir.5KG717100.v4.1 | 1.823 | 0.045077 | Protein of unknown function (DUF506) |
| Pavir.3KG017700.v4.1 | 1.8278 | 0.019488 | Calcium-binding EF-hand family protein |
| Pavir.2NG323200.v4.1 | 1.832 | 0.013867 | UDP-Glycosyltransferase / trehalose-phosphatase family protein |
| Pavir.7NG131800.v4.1 | 1.8518 | 0.013785 | SNF1-related protein kinase regulatory subunit gamma 1 |
| Pavir.3KG281700.v4.1 | 1.86 | 0.036716 | Bifunctional inhibitor/lipid-transfer protein/seed storage 2S albumin superfamily protein |
| Pavir.5KG244400.v4.1 | 1.8758 | 0.009368 | Peroxidase superfamily protein |
| Pavir.8KG004000.v4.1 | 1.8793 | 0.005642 | lipid transfer protein 12 |
| Pavir.5KG008300.v4.1 | 1.8943 | 0.027926 | ovate family protein 7 |
| Pavir.5NG214500.v4.1 | 1.9021 | 0.0264 | Adenine nucleotide alpha hydrolases-like superfamily protein |
| Pavir.9NG538800.v4.1 | 1.9062 | 0.035808 | serine carboxypeptidase-like 51 |
| Pavir.2NG323300.v4.1 | 1.9068 | 0.010771 | UDP-Glycosyltransferase / trehalose-phosphatase family protein |
| Pavir.9NG798900.v4.1 | 1.9138 | 0.010658 | Integrase-type DNA-binding superfamily protein |
| Pavir.3KG194100.v4.1 | 1.9166 | 0.039682 | NADP-malic enzyme 3 |
| Pavir.2KG219200.v4.1 | 1.924 | 0.034813 | RING domain ligase1 |
| Pavir.3NG317300.v4.1 | 1.9476 | 0.004771 | HXXXD-type acyl-transferase family protein |
| Pavir.3KG176100.v4.1 | 1.9794 | 0.007795 | Peroxidase superfamily protein |
| Pavir.8KG153400.v4.1 | 1.9868 | 0.028809 | cysteine-rich RLK (RECEPTOR-like protein kinase) 26 |
| Pavir.5KG195100.v4.1 | 2.0158 | 0.004372 | Integrase-type DNA-binding superfamily protein |
| Pavir.5KG154200.v4.1 | 2.0379 | 0.024891 | UDP-glucose:flavonoid 3-o-glucosyltransferase |
| Pavir.2KG436400.v4.1 | 2.0475 | 0.029311 |  |
| Pavir.5KG262600.v4.1 | 2.0578 | 0.002083 | Peroxidase superfamily protein |
| Pavir.5KG030900.v4.1 | 2.0727 | 0.003251 |  |
| Pavir.8KG167000.v4.1 | 2.0884 | 0.039902 | alcohol dehydrogenase 1 |
| Pavir.4NG172200.v4.1 | 2.0885 | 0.009982 | Eukaryotic aspartyl protease family protein |
| Pavir.2KG573600.v4.1 | 2.0964 | 0.018299 | NAD(P)-binding Rossmann-fold superfamily protein |
| Pavir.9NG654900.v4.1 | 2.1051 | 0.001192 | alpha/beta-Hydrolases superfamily protein |
| Pavir.1KG495000.v4.1 | 2.1064 | 0.003172 | aldehyde dehydrogenase 2B4 |
| Pavir.2KG030700.v4.1 | 2.1086 | 0.02745 | NAD(P)-binding Rossmann-fold superfamily protein |
| Pavir.2KG295300.v4.1 | 2.1121 | 0.004994 | Calcium-binding EF-hand family protein |
| Pavir.J015100.v4.1 | 2.1272 | 0.016421 | P-loop containing nucleoside triphosphate hydrolases superfamily protein |
| Pavir.8KG337600.v4.1 | 2.1404 | 0.015224 | cysteine-rich RLK (RECEPTOR-like protein kinase) 10 |
| Pavir.7NG042100.v4.1 | 2.1498 | 0.043923 | basic helix-loop-helix (bHLH) DNA-binding superfamily protein |
| Pavir.1KG480500.v4.1 | 2.157 | 0.014742 |  |
| Pavir.9KG538300.v4.1 | 2.163 | 0.002182 | hemoglobin 1 |
| Pavir.9KG386100.v4.1 | 2.167 | 0.009225 | cytochrome P450, family 78, subfamily A, polypeptide 5 |
| Pavir.9KG477600.v4.1 | 2.1682 | 0.000662 | asparagine synthetase 2 |
| Pavir.9KG652100.v4.1 | 2.17 | 0.009982 | Integrase-type DNA-binding superfamily protein |
| Pavir.2NG312700.v4.1 | 2.1791 | 0.011173 | Calcium-binding EF-hand family protein |
| Pavir.1KG131500.v4.1 | 2.1809 | 0.014047 | Cytochrome P450 superfamily protein |
| Pavir.9KG603900.v4.1 | 2.1899 | 0.033802 | Subtilase family protein |
| Pavir.3KG313200.v4.1 | 2.1947 | 0.003501 | WRKY family transcription factor |
| Pavir.3NG227100.v4.1 | 2.1965 | 0.028316 | ureide permease 4 |
| Pavir.4KG062200.v4.1 | 2.2079 | 0.029345 | 2-oxoglutarate (2OG) and Fe(II)-dependent oxygenase superfamily protein |
| Pavir.7NG422800.v4.1 | 2.2325 | 0.000447 | chloride channel A |
| Pavir.9KG134000.v4.1 | 2.2651 | 0.008502 |  |
| Pavir.3KG173900.v4.1 | 2.271 | 0.000446 | Peroxidase superfamily protein |
| Pavir.J785600.v4.1 | 2.2772 | 0.031022 | NB-ARC domain-containing disease resistance protein |
| Pavir.5NG037000.v4.1 | 2.2787 | 0.043425 | myb domain protein 87 |
| Pavir.2KG567600.v4.1 | 2.281 | 0.039902 | Adenine nucleotide alpha hydrolases-like superfamily protein |
| Pavir.4KG130300.v4.1 | 2.2912 | 0.019752 | Protein kinase superfamily protein |
| Pavir.2KG121700.v4.1 | 2.3197 | 0.019326 | Mannose-binding lectin superfamily protein |
| Pavir.5KG296000.v4.1 | 2.3199 | 0.000885 | ACC oxidase 1 |
| Pavir.7KG057500.v4.1 | 2.3295 | 0.001988 | ureide permease 4 |
| Pavir.2NG147500.v4.1 | 2.3351 | 0.0211 | WRKY DNA-binding protein 23 |
| Pavir.9NG335400.v4.1 | 2.3435 | 0.000441 |  |
| Pavir.5KG142700.v4.1 | 2.3826 | 0.003684 |  |
| Pavir.J650400.v4.1 | 2.3843 | 0.034806 |  |
| Pavir.J494900.v4.1 | 2.3852 | 0.018469 |  |
| Pavir.5KG648100.v4.1 | 2.3884 | 0.011173 | Late embryogenesis abundant (LEA) hydroxyproline-rich glycoprotein family |
| Pavir.6KG319400.v4.1 | 2.4077 | 0.000468 | Cupredoxin superfamily protein |
| Pavir.2KG242800.v4.1 | 2.4147 | 0.000952 | Bifunctional inhibitor/lipid-transfer protein/seed storage 2S albumin superfamily protein |
| Pavir.8KG059600.v4.1 | 2.4167 | 0.001058 | Ankyrin repeat family protein |
| Pavir.3NG227300.v4.1 | 2.4183 | 0.001259 | ureide permease 4 |
| Pavir.J431200.v4.1 | 2.4266 | 0.047406 |  |
| Pavir.5KG571800.v4.1 | 2.4342 | 0.039902 | Concanavalin A-like lectin protein kinase family protein |
| Pavir.1KG496300.v4.1 | 2.4414 | 0.000453 | aldehyde dehydrogenase 2B4 |
| Pavir.4NG126900.v4.1 | 2.4495 | 0.009729 |  |
| Pavir.6KG051700.v4.1 | 2.4651 | 0.043923 | tetratricopetide-repeat thioredoxin-like 3 |
| Pavir.2KG439000.v4.1 | 2.472 | 0.00794 |  |
| Pavir.8KG153100.v4.1 | 2.5149 | 0.000389 | cysteine-rich RLK (RECEPTOR-like protein kinase) 29 |
| Pavir.9NG495100.v4.1 | 2.5178 | 0.000376 | glutathione S-transferase TAU 18 |
| Pavir.3NG063400.v4.1 | 2.5482 | 0.02996 |  |
| Pavir.6KG408100.v4.1 | 2.5513 | 0.005069 | Jojoba acyl CoA reductase-related male sterility protein |
| Pavir.5NG117000.v4.1 | 2.5686 | 0.015174 |  |
| Pavir.3NG192200.v4.1 | 2.5737 | 0.019709 | sterol C4-methyl oxidase 1-2 |
| Pavir.8KG153500.v4.1 | 2.5822 | 0.003329 | HOPZ-ACTIVATED RESISTANCE 1 |
| Pavir.5NG345300.v4.1 | 2.5832 | 0.000141 | Peroxidase superfamily protein |
| Pavir.6KG166000.v4.1 | 2.5877 | 0.014537 |  |
| Pavir.5KG305800.v4.1 | 2.5971 | 0.034806 | Agenet domain-containing protein |
| Pavir.7NG106400.v4.1 | 2.6005 | 0.000351 | Chitinase family protein |
| Pavir.8NG245900.v4.1 | 2.6008 | 0.043118 | alcohol dehydrogenase 1 |
| Pavir.2NG615600.v4.1 | 2.6135 | 0.000129 | nicotianamine synthase 3 |
| Pavir.6KG038200.v4.1 | 2.6223 | 0.039983 | flavin-dependent monooxygenase 1 |
| Pavir.7KG032000.v4.1 | 2.6347 | 0.047659 | Transketolase |
| Pavir.5KG053800.v4.1 | 2.6641 | 0.045077 | myb domain protein 36 |
| Pavir.3KG509100.v4.1 | 2.6755 | 0.001426 | MLP-like protein 34 |
| Pavir.3KG261300.v4.1 | 2.6855 | 0.005446 | methionine sulfoxide reductase B 2 |
| Novel00722 | 2.7011 | 0.00018 | - |
| Pavir.3KG449800.v4.1 | 2.7179 | 0.001298 | Thioredoxin superfamily protein |
| Pavir.9KG573600.v4.1 | 2.7221 | 0.004332 | cytochrome P450, family 71, subfamily B, polypeptide 2 |
| Pavir.9KG132000.v4.1 | 2.7267 | 2.74E-05 |  |
| Pavir.7KG034300.v4.1 | 2.7389 | 0.021291 | Chitinase family protein |
| Pavir.3NG166800.v4.1 | 2.7472 | 1.62E-05 |  |
| Pavir.2NG031400.v4.1 | 2.7651 | 0.017056 | Concanavalin A-like lectin protein kinase family protein |
| Pavir.3KG449300.v4.1 | 2.7891 | 3.39E-05 | Thioredoxin superfamily protein |
| Pavir.8KG176800.v4.1 | 2.8354 | 0.001063 | Lateral organ boundaries (LOB) domain family protein |
| Pavir.6KG344400.v4.1 | 2.8575 | 0.018092 | wall associated kinase 3 |
| Pavir.6KG030300.v4.1 | 2.8716 | 3.72E-06 | Major facilitator superfamily protein |
| Pavir.2KG029500.v4.1 | 2.8747 | 0.003418 |  |
| Pavir.4KG178000.v4.1 | 2.8818 | 0.041799 |  |
| Pavir.8KG205100.v4.1 | 2.9017 | 0.029785 | Pyridoxal phosphate (PLP)-dependent transferases superfamily protein |
| Pavir.4NG040600.v4.1 | 2.9288 | 2.79E-05 | early nodulin-related |
| Pavir.9KG265100.v4.1 | 2.9746 | 0.028316 | GDSL-like Lipase/Acylhydrolase superfamily protein |
| Pavir.5KG487100.v4.1 | 3.0123 | 0.036551 | Protein kinase superfamily protein |
| Pavir.7KG358700.v4.1 | 3.024 | 0.016277 | receptor-like protein kinase 1 |
| Pavir.4KG397500.v4.1 | 3.0304 | 0.024559 | Phosphate-responsive 1 family protein |
| Pavir.6KG117500.v4.1 | 3.0446 | 0.030863 | Oxoglutarate/iron-dependent oxygenase |
| Pavir.7NG442500.v4.1 | 3.0477 | 0.032188 | Aluminium induced protein with YGL and LRDR motifs |
| Pavir.6NG087500.v4.1 | 3.0808 | 0.028778 |  |
| Pavir.5NG352300.v4.1 | 3.0996 | 0.002493 |  |
| Pavir.6NG145100.v4.1 | 3.1208 | 0.034806 | BURP domain-containing protein |
| Pavir.2NG116400.v4.1 | 3.1359 | 0.004788 | Late embryogenesis abundant (LEA) hydroxyproline-rich glycoprotein family |
| Pavir.4KG024500.v4.1 | 3.1438 | 4.02E-06 | early nodulin-related |
| Pavir.2NG648600.v4.1 | 3.1664 | 0.002423 | Peroxidase superfamily protein |
| Pavir.J286600.v4.1 | 3.1798 | 0.00464 | ammonium transporter 2 |
| Pavir.3KG237500.v4.1 | 3.1902 | 0.020194 | pinoresinol reductase 1 |
| Pavir.4NG151100.v4.1 | 3.2133 | 0.006687 | Ankyrin repeat family protein |
| Pavir.5NG054400.v4.1 | 3.2152 | 0.000177 | Protein of unknown function (DUF1637) |
| Pavir.6KG206800.v4.1 | 3.216 | 0.01475 |  |
| Pavir.8KG262000.v4.1 | 3.2573 | 0.00171 |  |
| Pavir.2KG260200.v4.1 | 3.2731 | 0.017585 |  |
| Pavir.2KG032100.v4.1 | 3.2927 | 0.00794 | Chalcone and stilbene synthase family protein |
| Pavir.J517000.v4.1 | 3.3219 | 0.001609 | cytochrome P450, family 72, subfamily A, polypeptide 15 |
| Pavir.1NG322900.v4.1 | 3.3429 | 9.58E-06 |  |
| Pavir.8KG246600.v4.1 | 3.3828 | 0.002212 |  |
| Novel00061 | 3.3939 | 0.041855 | - |
| Pavir.5KG180800.v4.1 | 3.44 | 0.002134 | Protein of unknown function (DUF594) |
| Pavir.3NG133700.v4.1 | 3.5526 | 3.5E-07 |  |
| Pavir.1KG216000.v4.1 | 3.5755 | 0.000173 |  |
| Pavir.5NG258500.v4.1 | 3.5818 | 5.96E-06 | ACC oxidase 1 |
| Pavir.1KG521900.v4.1 | 3.6314 | 0.005482 | Inorganic H pyrophosphatase family protein |
| Pavir.9NG290100.v4.1 | 3.6794 | 2.21E-07 | related to ABI3/VP1 2 |
| Pavir.8NG274300.v4.1 | 3.7024 | 0.01276 | beta-hydroxyisobutyryl-CoA hydrolase 1 |
| Pavir.7KG313400.v4.1 | 3.8024 | 0.041054 | 2-oxoglutarate (2OG) and Fe(II)-dependent oxygenase superfamily protein |
| Pavir.J277600.v4.1 | 3.8138 | 1.26E-07 | Late embryogenesis abundant (LEA) hydroxyproline-rich glycoprotein family |
| Pavir.9KG399300.v4.1 | 3.8378 | 5.97E-09 | organic cation/carnitine transporter 2 |
| Pavir.3KG227500.v4.1 | 3.8565 | 3.17E-07 |  |
| Pavir.2KG464500.v4.1 | 3.871 | 0.003061 | O-Glycosyl hydrolases family 17 protein |
| Pavir.8KG057700.v4.1 | 3.8743 | 0.032292 |  |
| Novel00269 | 3.8811 | 0.002334 | - |
| Pavir.J262800.v4.1 | 3.893 | 0.032324 | receptor-like protein kinase-related family protein |
| Pavir.3KG373400.v4.1 | 3.9137 | 0.008868 | NB-ARC domain-containing disease resistance protein |
| Pavir.5NG343000.v4.1 | 3.9555 | 6.52E-05 | LOB domain-containing protein 41 |
| Pavir.5NG022400.v4.1 | 3.9841 | 0.004295 | Ferritin/ribonucleotide reductase-like family protein |
| Pavir.5KG119000.v4.1 | 4.0289 | 0.001349 |  |
| Pavir.5KG119100.v4.1 | 4.1029 | 0.010346 | Cupredoxin superfamily protein |
| Pavir.2NG106300.v4.1 | 4.1394 | 0.039997 | Nucleic acid-binding, OB-fold-like protein |
| Pavir.1KG241700.v4.1 | 4.1718 | 7.92E-05 | cytochrome P450, family 76, subfamily C, polypeptide 4 |
| Pavir.1KG023400.v4.1 | 4.2489 | 0.00498 |  |
| Pavir.9NG529500.v4.1 | 4.2672 | 1.8E-06 |  |
| Pavir.3KG146600.v4.1 | 4.3081 | 6.67E-05 | Sucrose-6F-phosphate phosphohydrolase family protein |
| Pavir.9KG144800.v4.1 | 4.3222 | 0.002065 | nodulin MtN21 /EamA-like transporter family protein |
| Pavir.1KG393200.v4.1 | 4.3589 | 0.001628 | wall associated kinase 5 |
| Pavir.7KG024800.v4.1 | 4.3988 | 1.4E-07 | expansin 11 |
| Pavir.9NG738300.v4.1 | 4.4123 | 4.44E-06 | P-loop containing nucleoside triphosphate hydrolases superfamily protein |
| Pavir.9KG470200.v4.1 | 4.4494 | 2.06E-11 | Protein of unknown function (DUF1262) |
| Pavir.6KG138500.v4.1 | 4.4512 | 0.000548 | cytochrome P450, family 71, subfamily A, polypeptide 22 |
| Pavir.7NG175500.v4.1 | 4.4752 | 0.000778 | AGAMOUS-like 16 |
| Pavir.2KG242600.v4.1 | 4.4969 | 2.07E-07 | Bifunctional inhibitor/lipid-transfer protein/seed storage 2S albumin superfamily protein |
| Pavir.4NG301100.v4.1 | 4.5047 | 0.000971 | Ankyrin repeat family protein |
| Pavir.9NG387900.v4.1 | 4.512 | 0.005029 |  |
| Pavir.7NG071900.v4.1 | 4.5448 | 0.000619 | cysteine-rich RLK (RECEPTOR-like protein kinase) 6 |
| Pavir.4KG380000.v4.1 | 4.5725 | 0.000111 |  |
| Pavir.5KG285600.v4.1 | 4.7145 | 0.001561 | HXXXD-type acyl-transferase family protein |
| Pavir.2NG266200.v4.1 | 4.7484 | 0.000502 | Bifunctional inhibitor/lipid-transfer protein/seed storage 2S albumin superfamily protein |
| Pavir.7NG417800.v4.1 | 4.7554 | 5.22E-08 | Peroxidase superfamily protein |
| Pavir.4KG371300.v4.1 | 4.7765 | 0.000114 |  |
| Pavir.4KG032900.v4.1 | 4.8081 | 0.000214 |  |
| Pavir.4KG114700.v4.1 | 4.8589 | 0.01444 | basic chitinase |
| Pavir.7KG379200.v4.1 | 4.8619 | 0.014358 | thionin 2.2 |
| Pavir.6KG344100.v4.1 | 4.8644 | 0.014298 | global transcription factor group E4 |
| Pavir.9NG444900.v4.1 | 4.8656 | 0.024669 | Late embryogenesis abundant (LEA) hydroxyproline-rich glycoprotein family |
| Pavir.2KG188600.v4.1 | 4.9474 | 2.9E-09 |  |
| Pavir.9NG180000.v4.1 | 5.1505 | 0.001228 | Protein of unknown function (DUF1637) |
| Pavir.2KG309000.v4.1 | 5.2326 | 7.99E-05 |  |
| Pavir.7KG258900.v4.1 | 5.2367 | 0.046474 |  |
| Pavir.5NG125600.v4.1 | 5.2702 | 0.010035 | LOB domain-containing protein 41 |
| Pavir.2NG095900.v4.1 | 5.4183 | 0.040988 |  |
| Pavir.8KG393500.v4.1 | 5.4302 | 5.98E-15 | cysteine-rich RLK (RECEPTOR-like protein kinase) 25 |
| Novel00171 | 5.4425 | 3.76E-14 | - |
| Pavir.4NG040700.v4.1 | 5.4535 | 1.65E-11 | early nodulin-related |
| Pavir.5KG242100.v4.1 | 5.6596 | 0.015224 |  |
| Pavir.3NG186400.v4.1 | 5.7701 | 0.006504 | Thioredoxin superfamily protein |
| Pavir.4KG026900.v4.1 | 5.7911 | 8.86E-06 | early nodulin-related |
| Pavir.1NG162100.v4.1 | 5.797 | 5.71E-09 |  |
| Pavir.8KG320500.v4.1 | 5.848 | 1.41E-07 | cysteine-rich RLK (RECEPTOR-like protein kinase) 28 |
| Pavir.5NG287200.v4.1 | 5.8529 | 7.64E-16 | beta glucosidase 17 |
| Pavir.8NG283000.v4.1 | 5.8571 | 0.005482 |  |
| Pavir.2NG301700.v4.1 | 6.019 | 1.57E-10 |  |
| Novel00298 | 6.0296 | 4.02E-06 | - |
| Pavir.4KG008800.v4.1 | 6.2037 | 0.004452 |  |
| Pavir.2KG586900.v4.1 | 6.2664 | 0.001202 | WRKY DNA-binding protein 54 |
| Pavir.9KG315400.v4.1 | 6.304 | 2.01E-05 | basic chitinase |
| Pavir.J438200.v4.1 | 6.3464 | 0.000281 | NB-ARC domain-containing disease resistance protein |
| Pavir.6KG027400.v4.1 | 6.4377 | 0.000157 | glycerol-3-phosphate acyltransferase 7 |
| Pavir.9KG652300.v4.1 | 6.5472 | 0.012377 | Integrase-type DNA-binding superfamily protein |
| Pavir.J134800.v4.1 | 6.6859 | 1.36E-13 | beta glucosidase 12 |
| Pavir.5KG116900.v4.1 | 6.9689 | 1E-05 | LOB domain-containing protein 41 |
| Novel00363 | 7.4511 | 1.94E-24 | - |
| Pavir.5NG184700.v4.1 | 11.227 | 1.99E-34 |  |
| Pavir.6NG345600.v4.1 | 11.253 | 1.78E-34 |  |
| Novel00062 | Inf | 0.033946 | - |
| Novel00559 | Inf | 1.52E-14 | - |
| Novel00582 | Inf | 3.76E-07 | - |
| Novel00742 | Inf | 0.002554 | - |
| Novel01214 | Inf | 1.99E-16 | - |
| Novel01318 | Inf | 0.003064 | - |
| Novel01326 | Inf | 1.52E-14 | - |
| Novel01328 | Inf | 0.000109 | - |
| Novel01367 | Inf | 0.003061 | - |
| Pavir.2NG266000.v4.1 | Inf | 5.77E-05 | Bifunctional inhibitor/lipid-transfer protein/seed storage 2S albumin superfamily protein |
| Pavir.8KG391800.v4.1 | Inf | 0.020875 | C2H2 and C2HC zinc fingers superfamily protein |
| Pavir.9KG355200.v4.1 | Inf | 5.5E-06 | cysteine-rich RLK (RECEPTOR-like protein kinase) 39 |
| Pavir.2KG554700.v4.1 | Inf | 0.004112 | Lung seven transmembrane receptor family protein |
| Pavir.9NG738400.v4.1 | Inf | 0.004135 | P-loop containing nucleoside triphosphate hydrolases superfamily protein |
| Pavir.6KG027300.v4.1 | Inf | 7.26E-06 | TTF-type zinc finger protein with HAT dimerisation domain |
| Pavir.1NG130700.v4.1 | Inf | 3.05E-08 |  |
| Pavir.1NG317600.v4.1 | Inf | 0.029146 |  |
| Pavir.3KG001700.v4.1 | Inf | 0.041152 |  |
| Pavir.3KG290200.v4.1 | Inf | 0.018092 |  |
| Pavir.3NG260000.v4.1 | Inf | 0.001213 |  |
| Pavir.3NG260100.v4.1 | Inf | 0.000168 |  |
| Pavir.4KG354900.v4.1 | Inf | 1.65E-08 |  |
| Pavir.5NG022500.v4.1 | Inf | 7.92E-05 |  |
| Pavir.5NG267500.v4.1 | Inf | 0.019416 |  |
| Pavir.8KG195200.v4.1 | Inf | 0.018508 |  |
| Pavir.8NG032100.v4.1 | Inf | 0.047216 |  |
| Pavir.9KG067200.v4.1 | Inf | 0.034209 |  |
| Pavir.J423400.v4.1 | Inf | 2.01E-05 |  |
| Novel00067 | #NAME? | 4.47E-12 | - |
| Novel01340 | #NAME? | 7.36E-10 | - |
| Novel00712 | #NAME? | 2.73E-06 | - |
| Novel01278 | #NAME? | 3.04E-06 | - |
| Novel00692 | #NAME? | 0.001228 | - |
| Novel00576 | #NAME? | 0.002724 | - |
| Novel00704 | #NAME? | 0.003119 | - |
| Novel00807 | #NAME? | 0.006391 | - |
| Novel00671 | #NAME? | 0.010559 | - |
| Pavir.5NG541500.v4.1 | #NAME? | 0.04762 | alpha/beta-Hydrolases superfamily protein |
| Pavir.5NG632900.v4.1 | #NAME? | 0.000516 | basic helix-loop-helix (bHLH) DNA-binding superfamily protein |
| Pavir.J490700.v4.1 | #NAME? | 0.016267 | BED zinc finger ;hAT family dimerisation domain |
| Pavir.J095600.v4.1 | #NAME? | 2.53E-13 | beta-glucosidase 45 |
| Pavir.5KG686700.v4.1 | #NAME? | 0.008914 | Calcineurin-like metallo-phosphoesterase superfamily protein |
| Pavir.5NG610200.v4.1 | #NAME? | 0.012176 | calcium-dependent protein kinase 13 |
| Pavir.7KG281100.v4.1 | #NAME? | 0.008868 | cyclin p3;1 |
| Pavir.J013400.v4.1 | #NAME? | 4.97E-07 | cytochrome p450 71b6 |
| Pavir.5NG225300.v4.1 | #NAME? | 0.000286 | cytochrome P450, family 71, subfamily B, polypeptide 20 |
| Pavir.5NG225200.v4.1 | #NAME? | 7.38E-07 | don-glucosyltransferase 1 |
| Pavir.7KG233800.v4.1 | #NAME? | 0.0264 | Eukaryotic aspartyl protease family protein |
| Pavir.5NG493500.v4.1 | #NAME? | 0.012079 | glyceraldehyde-3-phosphate dehydrogenase C2 |
| Pavir.7KG041700.v4.1 | #NAME? | 2.35E-09 | Helicase protein with RING/U-box domain |
| Pavir.5NG564900.v4.1 | #NAME? | 0.008244 | histone acetyltransferase of the MYST family 2 |
| Pavir.7KG262500.v4.1 | #NAME? | 0.021441 | Leucine-rich repeat protein kinase family protein |
| Pavir.J534500.v4.1 | #NAME? | 5.32E-09 | LRR and NB-ARC domains-containing disease resistance protein |
| Pavir.J534400.v4.1 | #NAME? | 0.00025 | NB-ARC domain-containing disease resistance protein |
| Pavir.5NG461000.v4.1 | #NAME? | 0.000646 | origin recognition complex subunit 4 |
| Pavir.5NG579900.v4.1 | #NAME? | 0.000208 | Plant protein 1589 of unknown function |
| Pavir.7KG207100.v4.1 | #NAME? | 0.000238 | protein-l-isoaspartate methyltransferase 1 |
| Pavir.5NG516100.v4.1 | #NAME? | 1.79E-06 | Radical SAM superfamily protein |
| Pavir.7KG014900.v4.1 | #NAME? | 7.3E-06 | serine carboxypeptidase-like 20 |
| Pavir.5NG609100.v4.1 | #NAME? | 3.37E-05 | TBP-associated factor 12 |
| Pavir.4KG112100.v4.1 | #NAME? | 3.5E-05 | U2 snRNP auxilliary factor, large subunit, splicing factor |
| Pavir.3KG455400.v4.1 | #NAME? | 5.32E-09 | Ubiquinol-cytochrome C reductase hinge protein |
| Pavir.7KG233300.v4.1 | #NAME? | 5.36E-08 | VIER F-box proteine 1 |
| Pavir.7KG053800.v4.1 | #NAME? | 2.27E-14 |  |
| Pavir.6NG272200.v4.1 | #NAME? | 5.29E-14 |  |
| Pavir.7KG204000.v4.1 | #NAME? | 1.33E-09 |  |
| Pavir.5NG552700.v4.1 | #NAME? | 9.73E-09 |  |
| Pavir.8KG183000.v4.1 | #NAME? | 1.1E-08 |  |
| Pavir.7KG173900.v4.1 | #NAME? | 1.77E-07 |  |
| Pavir.5KG713500.v4.1 | #NAME? | 2.91E-07 |  |
| Pavir.7KG239700.v4.1 | #NAME? | 1.07E-06 |  |
| Pavir.5NG243500.v4.1 | #NAME? | 1.22E-06 |  |
| Pavir.7KG219600.v4.1 | #NAME? | 1.36E-06 |  |
| Pavir.4NG228100.v4.1 | #NAME? | 7.71E-05 |  |
| Pavir.7KG007700.v4.1 | #NAME? | 0.000414 |  |
| Pavir.5NG557300.v4.1 | #NAME? | 0.000443 |  |
| Pavir.5NG540500.v4.1 | #NAME? | 0.000733 |  |
| Pavir.4KG163900.v4.1 | #NAME? | 0.001967 |  |
| Pavir.5NG236100.v4.1 | #NAME? | 0.004197 |  |
| Pavir.5NG379200.v4.1 | #NAME? | 0.004776 |  |
| Pavir.7KG369600.v4.1 | #NAME? | 0.00765 |  |
| Pavir.6NG265400.v4.1 | #NAME? | 0.014664 |  |
| Pavir.7KG129000.v4.1 | #NAME? | 0.021291 |  |
| Pavir.5NG302000.v4.1 | #NAME? | 0.022607 |  |
| Pavir.5NG493800.v4.1 | #NAME? | 0.035531 |  |
| Pavir.5NG648600.v4.1 | #NAME? | 0.03887 |  |

**Sheet 2**

| **Pathway** | **Gene_id** | **log2FoldChange** | **q-value** | **GeneDescription** |
| --- | --- | --- | --- | --- |
| Amino acid metabolite | Pavir.9KG477600.v4.1 | 2.1682 | 2.3E-06 | asparagine synthetase 2 (ASNS) |
|  | Pavir.5NG406700.v4.1 | -1.9691 | 0.003789 | ketol-acid reductoisomerase |
|  | Pavir.7KG177700.v4.1 | -1.842 | 0.012305 | glutamate decarboxylase 2 (GDH) |
|  | Pavir.5NG415700.v4.1 | -1.5605 | 0.047216 | NADH-dependent glutamate synthase 1 (GOGAT) |
|  | Pavir.6KG328800.v4.1 | -2.7557 | 0.001621 | GLN phosphoribosyl pyrophosphate amidotransferase 1 (GPAT) |
|  | Pavir.8NG274300.v4.1 | 3.7024 | 0.01276 | beta-hydroxyisobutyryl-CoA hydrolase 1 |
|  | Pavir.7KG268800.v4.1 | -2.0276 | 0.003083 | aldehyde dehydrogenase 3F1 |
|  | Pavir.1KG496300.v4.1 | 2.4414 | 0.000453 | aldehyde dehydrogenase 2B4 |
|  | Pavir.1KG495000.v4.1 | 2.1064 | 0.003172 | aldehyde dehydrogenase 2B4 |
|  | Pavir.7KG014900.v4.1 | ∞ | 7.3E-06 | serine carboxypeptidase-like 20 |
|  | Pavir.5NG575400.v4.1 | -2.0186 | 0.034516 | serine carboxypeptidase-like 27 |
|  | Pavir.9NG538800.v4.1 | 1.9062 | 0.035808 | serine carboxypeptidase-like 51 |
|  | Pavir.J354700.v4.1 | -1.9147 | 0.007877 | serine hydroxymethyltransferase 7 |
|  | Pavir.5NG528800.v4.1 | -2.797 | 0.046474 | serine-rich protein-related |
| C1 metabolite | Pavir.5NG271300.v4.1 | -2.9667 | 0.028037 | Cystathionine beta-synthase (CBS) protein |
| Lignin biosynthesis | Pavir.5KG262600.v4.1 | 2.0578 | 0.002083 | Peroxidase superfamily protein 42 |
|  | Pavir.5KG244400.v4.1 | 1.8758 | 0.009368 | Peroxidase superfamily protein 66 |
|  | Pavir.9NG509200.v4.1 | 1.7478 | 0.020555 | Peroxidase superfamily protein 70 |
|  | Pavir.7NG417800.v4.1 | 4.7554 | 5.22E-08 | Peroxidase superfamily protein 6 |
|  | Pavir.3KG173900.v4.1 | 2.271 | 0.000446 | Peroxidase superfamily protein 73 |
|  | Pavir.3KG176100.v4.1 | 1.9794 | 0.007795 | Peroxidase superfamily protein 52 |
|  | Pavir.5NG345300.v4.1 | 2.5832 | 0.000141 | Peroxidase superfamily protein 66 |
|  | Pavir.2NG648600.v4.1 | 3.1664 | 0.002423 | Peroxidase superfamily protein 66 |
| Phenylpropanoid biosynthesis | Pavir.J095600.v4.1 | ∞ | 2.53E-13 | beta-glucosidase 45 |
|  | Pavir.J134800.v4.1 | 6.6859 | 1.36E-13 | beta glucosidase 12 |
|  | Pavir.5NG287200.v4.1 | 5.8529 | 7.64E-16 | beta glucosidase 17 |
|  | Pavir.3NG182300.v4.1 | -8.9163 | 5.22E-18 | beta glucosidase 42 |
|  | Pavir.2KG573600.v4.1 | 2.0964 | 0.018299 | NAD(P)-binding Rossmann-fold superfamily protein |
|  | Pavir.2KG030700.v4.1 | 2.1086 | 0.02745 | NAD(P)-binding Rossmann-fold superfamily protein |
|  | Pavir.7KG008000.v4.1 | -3.243 | 0.002081 | NAD(P)-linked oxidoreductase superfamily protein |
|  | Pavir.5KG615300.v4.1 | -2.132 | 0.006391 | NAD(P)-linked oxidoreductase superfamily protein |
|  | Pavir.5NG415700.v4.1 | -1.5605 | 0.047216 | NADH-dependent glutamate synthase 1 |
|  | Pavir.5NG588800.v4.1 | -1.615 | 0.035899 | NADH-ubiquinone oxidoreductase 20 kDa subunit, mitochondrial |
|  | Pavir.5NG473400.v4.1 | -3.2091 | 7.23E-08 | NADP-malic enzyme 3 |
|  | Pavir.3KG194100.v4.1 | 1.9166 | 0.039682 | NADP-malic enzyme 3 |
|  | Pavir.5NG553300.v4.1 | -1.871 | 0.007376 | cytochrome P450, family 98, subfamily A, polypeptide 3 |
|  | Pavir.5KG154200.v4.1 | 2.0379 | 0.024891 | UDP-glucose:flavonoid 3-o-glucosyltransferase |
|  | Pavir.6NG171100.v4.1 | -2.3364 | 0.000673 | UDP-glucosyl transferase 73B5 |
|  | Pavir.7KG141500.v4.1 | -6.5015 | 0.006369 | UDP-glucosyl transferase 85A2 |
|  | Pavir.7KG297500.v4.1 | -2.9085 | 0.000168 | UDP-glucosyl transferase 85A2 |
|  | Pavir.5NG482300.v4.1 | -4.717 | 2.01E-05 | UDP-glucosyl transferase 88A1 |
|  | Pavir.5NG481800.v4.1 | -2.4708 | 0.002542 | UDP-glucosyl transferase 88A1 |
|  | Pavir.2NG323200.v4.1 | 1.832 | 0.013867 | UDP-Glycosyltransferase / trehalose-phosphatase family protein |
|  | Pavir.2NG323300.v4.1 | 1.9068 | 0.010771 | UDP-Glycosyltransferase / trehalose-phosphatase family protein |
|  | Pavir.5KG656500.v4.1 | -2.038 | 0.035487 | UDP-Glycosyltransferase superfamily protein |
| Hormone | Pavir.5NG554100.v4.1 | -2.548 | 0.001616 | ABA-responsive element binding protein 3 (ABF) |
|  | Pavir.5NG613200.v4.1 | -1.9364 | 0.004598 | auxin response factor 2 (ARF) |
|  | Pavir.5NG558600.v4.1 | -1.7684 | 0.019737 | Auxin efflux carrier family protein |
|  | Pavir.5NG590200.v4.1 | -1.6173 | 0.037255 | Leucine-rich receptor-like protein kinase family protein |
|  | Pavir.5NG636300.v4.1 | -2.3922 | 0.000171 | Leucine-rich receptor-like protein kinase family protein |
|  | Pavir.5NG430100.v4.1 | -1.9264 | 0.011845 | indoleacetic acid-induced protein 8 (IAA6-like) |
|  | Pavir.3KG477400.v4.1 | -7.1282 | 1.36E-07 |  |
|  | Pavir.3KG477800.v4.1 | -2.0167 | 0.017464 | response regulator 11 |
|  | Pavir.5NG425700.v4.1 | -1.7065 | 0.031299 | Transcriptional factor B3 family protein / auxin-responsive factor  AUX/IAA-related (B-ARR) |
|  | Pavir.8KG360600.v4.1 | -2.472 | 0.002212 | Transmembrane amino acid transporter family protein |
|  | Pavir.5KG296000.v4.1 | 2.3199 | 0.000885 | ACC oxidase 1 (ACO) |
|  | Pavir.5NG258500.v4.1 | 3.5818 | 5.96E-06 | ACC oxidase 1 (ACO) |
|  | Pavir.5KG065800.v4.1 | -2.134 | 0.004033 | gibberellin 3-oxidase 1 (GAI) |
|  | Pavir.7KG194800.v4.1 | -4.9654 | 1.26E-07 | Gibberellin-regulated family protein |
| methyltransferase | Pavir.5NG587200.v4.1 | -3.1618 | 0.001576 | S-adenosyl-L-methionine-dependent methyltransferases superfamily protein |
|  | Pavir.5NG109300.v4.1 | -3.1175 | 0.008152 | S-adenosyl-L-methionine-dependent methyltransferases superfamily protein |
|  | Pavir.7KG242900.v4.1 | -3.1013 | 0.003822 | S-adenosyl-L-methionine-dependent methyltransferases superfamily protein |
|  | Pavir.6KG121500.v4.1 | -2.9583 | 0.010507 | S-adenosyl-L-methionine-dependent methyltransferases superfamily protein |
|  | Pavir.5NG452100.v4.1 | -2.4881 | 0.00033 | S-adenosyl-L-methionine-dependent methyltransferases superfamily protein |
|  | Pavir.5NG587300.v4.1 | -2.0917 | 0.02708 | S-adenosyl-L-methionine-dependent methyltransferases superfamily protein |
|  | Pavir.1NG233900.v4.1 | -1.8138 | 0.013035 | S-adenosyl-L-methionine-dependent methyltransferases superfamily protein |
| Transcription factors | Pavir.5NG589800.v4.1 | -1.9054 | 0.038191 | Alcohol dehydrogenase transcription factor Myb/SANT-like family protein |
|  | Pavir.5NG613200.v4.1 | -1.9364 | 0.004598 | auxin response factor 2 |
|  | Pavir.7KG064900.v4.1 | -8.45 | 2.52E-06 | basic helix-loop-helix (bHLH) DNA-binding superfamily protein |
|  | Pavir.6NG278200.v4.1 | -3.3549 | 0.016455 | basic helix-loop-helix (bHLH) DNA-binding superfamily protein |
|  | Pavir.5NG613400.v4.1 | -2.9346 | 0.000208 | basic helix-loop-helix (bHLH) DNA-binding superfamily protein |
|  | Pavir.5NG632900.v4.1 | ∞ | 0.000516 | basic helix-loop-helix (bHLH) DNA-binding superfamily protein |
|  | Pavir.5NG502600.v4.1 | -4.4765 | 2.26E-11 | Basic-leucine zipper (bZIP) transcription factor family protein |
|  | Pavir.5NG548700.v4.1 | -5.4162 | 0.000252 | F-box family protein |
|  | Pavir.7NG030500.v4.1 | -5.292 | 7.93E-06 | F-box family protein |
|  | Pavir.5NG548200.v4.1 | -2.0269 | 0.026404 | myb domain protein 33 |
|  | Pavir.J313400.v4.1 | -2.1485 | 0.001628 | myb domain protein 86 |
|  | Pavir.5NG500800.v4.1 | -3.5388 | 0.000823 | Transducin/WD40 repeat-like superfamily protein |
|  | Pavir.5NG459100.v4.1 | -2.6642 | 1.86E-05 | Transducin/WD40 repeat-like superfamily protein |
|  | Pavir.5NG617400.v4.1 | -1.7083 | 0.031238 | Transducin/WD40 repeat-like superfamily protein |
|  | Pavir.5NG037000.v4.1 | 2.2787 | 0.043425 | myb domain protein 87 |
|  | Pavir.5KG053800.v4.1 | 2.6641 | 0.045077 | myb domain protein 36 |
|  | Pavir.2NG147500.v4.1 | 2.3351 | 0.0211 | WRKY DNA-binding protein 23 |
|  | Pavir.2KG586900.v4.1 | 6.2664 | 0.001202 | WRKY DNA-binding protein 54 |
|  | Pavir.3KG313200.v4.1 | 2.1947 | 0.003501 | WRKY family transcription factor |
|  | Pavir.5NG501200.v4.1 | -1.9996 | 0.004288 | Transcriptional factor B3 family protein / auxin-responsive factor  AUX/IAA-related |
|  | Pavir.5NG425700.v4.1 | -1.7065 | 0.031299 | Transcriptional factor B3 family protein / auxin-responsive factor  AUX/IAA-related |

**Table S4.** Primers used in this study.

| Primer name | Sequence (5’- 3’) |
| --- | --- |
| For gene cloning |  |
| PvCGS.F | CCCTAGCTCCTTCTCCTCAGC |
| PvCGS.R | ATTAAAGTGAACGAATCAAGTGCT |
| PvSAHH1.F | ACTCCCAATCCAATCCGCGAG |
| PvSAHH1.R | GGTGTTCTAGTTGTGCTGGGC |
| For qRT-PCR analysis |  |
| PvCGS.qF | GAGATCTGTGAGAATATTTGAGCCG |
| PvCGS.qR | CGAGACTGCAGAGTACAGAGGTCAA |
| PvSAHH.qF | GAAGTTAGCCACATCGGGTTATCTT |
| PvSAHH.qR | ACTCAACCAAACTAAACCGCTCAAG |
| PvUBQ.F | TTCGTGGTGGCCAGTAAG |
| PvUBQ.R | AGAGACCAGAAGACCCAGGTACAG |
| For RNAi vector construction |  |
| PvCGSRi.F | AAACTTCGTGCGCCAGCTCAG |
| PvCGSRi.R | CGAAACTCCCCTCTTAGGGAG |
| PvSAHHRi.F | CTGTGCCCTTCAAGCCCTGA |
| PvSAHHRi.R | CCAAGCTTGCCCAAGTGGAGA |
| For transgenic plants identification | |
| PvCGSRi.gF | CGTCGTCGGTGAACAGGTAT |
| PvCGSRi.gR | TAGACCTTCTCAAGGGCCTGA |
| PvSAHHRi.gF | CGTCGTCGGTGAACAGGTAT |
| PvSAHHRi.gR | ATGCCAGTCTTGGTCTCAGGG |
| Guslinker.F | AACAGTTCCTGATTAACCACAAACC |
| Guslinker.R | GCCAGAAGTTCTTTTTCCAGTACC |
| Hph3 | AAGGAATCGGTCAATACACTACATGG |
| Hph4 | AAGACCAATGCGGAGCATATACG |
